# ZBP1‐mediated Treg cell dysfunction exacerbates neuroimmune dysregulation in Alzheimer's disease under metabolic stress

**DOI:** 10.1002/alz.71896

**Published:** 2026-09-30

**Authors:** Shanshan Huang, Zengyu Zhang, Jiajie Xia, Mengqiu Deng, Fangzheng Chen, Meng Wang, Yuchao Fei, Zhiwen Jiang, Chenyu Lu, Jiayu Fu, Jingchun Guo, Houguang Zhou

**Affiliations:** ^1^ Department of Geriatrics National Clinical Research Center for Aging and Medicine Huashan Hospital, Fudan University Shanghai China; ^2^ MOE Frontiers Center for Brain Science Institutes of Brain Science Department of Translational Neuroscience Jing'an District Centre Hospital of Shanghai Fudan University Shanghai China; ^3^ Department of Endocrinology Affiliated Hospital of Jiangnan University Jiangnan University Wuxi China; ^4^ Department of Rehabilitation Medicine Huashan Hospital, Fudan University Shanghai China; ^5^ Department of Neurosurgery Huashan Hospital, Fudan University Shanghai China; ^6^ Department of Anesthesiology Changzheng Hospital Second Affiliated Hospital of Naval Medical University Shanghai China

**Keywords:** Alzheimer's disease, metabolic stress, neuroimmune, regulatory T cells, Z‐DNA‐binding protein 1

## Abstract

**INTRODUCTION:**

Metabolic stress increases Alzheimer's disease (AD) risk, but mechanisms linking metabolic dysfunction to neuroimmune dysregulation remain unclear.

**METHODS:**

We assessed cognition in community‐dwelling participants and used high‐fat diet (HFD)‐fed 5xFAD mice, high glucose and high palmitate (HGHP)‐treated regulatory T cell (Treg) co‐cultures, and adoptive Treg transfer to evaluate metabolic stress‐associated neuroimmune dysfunction. Flow cytometry, RNA sequencing, positron emission tomography computed tomography, and electrophysiology assessed Treg function, AD pathology, cerebral metabolism, and synaptic plasticity.

**RESULTS:**

Metabolic stress impaired Treg stability and function, accompanied by increased Z‐DNA‐binding protein 1 (ZBP1) expression and activation of apoptotic and necroptotic signaling. Treg dysfunction was associated with enhanced glial activation and neuronal injury. Under HGHP stress, ZBP1‐deficient Tregs showed improved survival and stronger immunosuppressive activity, more effectively limiting glial activation and preserving neuronal integrity. In vivo, ZBP1‐deficient Treg transfer conferred greater neuroprotection than wild‐type Treg transfer in HFD‐fed AD mice.

**DISCUSSION:**

ZBP1 may contribute to metabolic stress–induced Treg dysfunction and represent a candidate target to improve Treg‐based therapy in HFD‐fed AD mice.

## Highlights

- Metabolic stress impairs the stability and immunosuppressive function of regulatory T cells (Tregs) in Alzheimer's disease (AD).
- Z‐DNA‐binding protein 1 (ZBP1) expression is upregulated in Treg cells under metabolic stress and is associated with cell death–related signaling.
- ZBP1 deficiency preserves Treg‐cell function and suppresses neuroinflammatory responses under glucolipotoxic conditions.
- Adoptive transfer of ZBP1‐deficient Treg cells improves pathology and synaptic function in high‐fat diet–fed AD mice.

## 1 BACKGROUND

Alzheimer's disease (AD) is a neurodegenerative disorder characterized by cognitive decline, synaptic dysfunction, and amyloid beta (Aβ) accumulation.^1^, ^2^ Although aging and genetic susceptibility remain the major risk factors, increasing evidence suggests that metabolic disturbance also influences AD risk and progression.^3^, ^4^ Epidemiological studies have shown that individuals with type 2 diabetes mellitus (T2DM) and obesity are at increased risk of cognitive decline and dementia.^5^, ^6^, ^7^ Meanwhile, experimental studies have shown that a high‐fat diet (HFD) worsens Aβ pathology, gliosis, and cognitive impairment in AD mouse models.^4^, ^8^ These findings suggest that metabolic abnormalities are not only common comorbidities but may also contribute directly to disease progression. However, the mechanisms by which metabolic stress worsens AD remain unclear.

Accumulating evidence indicates that metabolic stress alters the peripheral immune system and, in turn, affects immune regulation in the brain.^9^, ^10^ Given the blood–brain barrier, HFD or glucolipotoxic stress‐induced metabolic perturbations likely act first on peripheral tissues, especially immune cells sensitive to nutrient, lipid, and inflammatory cues.^11^, ^12^ AD is now increasingly viewed as a disease influenced by peripheral immune changes.^13^, ^14^ Peripheral immune cells can shape central nervous system (CNS) homeostasis and disease progression through sustained immune‐to‐brain communication.^15^, ^16^ Among peripheral immune populations, regulatory T cells (Tregs) have attracted particular attention for their central role in preserving immune tolerance and constraining aberrant inflammatory responses.^17^, ^18^ Under pathological conditions, Tregs can enter the CNS and influence glial activation, neuronal survival, and synaptic integrity.^19^ In models of neurodegeneration, expansion or adoptive transfer of Tregs has been shown to reduce neuroinflammation and delay disease progression, supporting protective roles for Tregs in the nervous system.^20^, ^21^, ^22^ These observations suggest that impaired Treg‐mediated immune regulation may contribute to persistent glial activation and neuronal damage. However, whether metabolic stress affects Treg stability and function in AD remains poorly understood.

Z‐DNA‐binding protein 1 (ZBP1) may be a plausible mediator of this process. ZBP1 is a cytosolic nucleic‐acid sensor involved in inflammatory signaling and regulated cell death.^23^, ^24^ It responds to stress and danger signals by activating apoptosis, necroptosis, and pyroptosis through receptor interacting serine/threonine kinase 3 (RIPK3)‐ and caspase‐dependent pathways, thereby contributing to the broader process of PANoptosis.^25^ Although ZBP1 has been largely studied in innate immune settings, particularly in infection and inflammation,^26^ its role in adaptive immune cells is still not well defined. Profiling studies indicate prominent ZBP1 expression throughout T‐cell populations, showing enrichment in Treg subsets,^27^, ^28^ which raises the possibility that ZBP1 may affect both the survival and the immunoregulatory function of Tregs under metabolic stress, and thereby contribute to neuroimmune changes relevant to AD progression.

With these considerations, we hypothesized that long‐term metabolic stress activates ZBP1 in Tregs, thereby impairing Treg survival and immunoregulatory function and promoting neuroimmune dysregulation in AD. In this study, we investigated the role of ZBP1 in Treg dysfunction under metabolic stress and examined how this interaction influences glial activation, neuronal injury, and cognitive impairment progression. This study will clarify how metabolic disturbance affects the peripheral immune–brain axis and evaluates the contribution of ZBP1 in AD therapy.

## 2 METHODS

### 2.1 Participants

Participants in this study were drawn from the Shanghai Community Health Survey cohort established by Huashan Hospital. This multicenter cross‐sectional study was conducted from September 2023 to May 2024, using multistage cluster random sampling to select residents aged ≥ 65 years from eight communities in Shanghai as participants in the health survey. Participants were: (1) individuals aged ≥ 65 years who signed informed consent forms and agreed to participate in the survey; (2) older residents living within the study area, defined as elderly people living independently in a specific community or neighborhood, excluding living in institutions such as hospitals, nursing homes, or assisted living facilities; (3) older people screened for cognitive impairment using the Mini‐Mental State Examination (MMSE). Exclusion criteria were: (1) incomplete responses to the MMSE and 9‐item Subjective Cognitive Decline Questionnaire (SCD‐Q9) or (2) the presence of diseases that may significantly affect cognitive function (Parkinson's disease; a diagnosis of severe mental illness; self‐reported symptoms of dizziness and headache or a history of stroke; a history of fracture, trauma, or surgery within the past 3 months; the presence of malignant tumors). The final analysis included 290 participants with cognitive impairments, which was grouped into: T2DM group (*n =* 102) and non‐T2DM group (*n =* 188). This study protocol has been approved by the ethics committee of Huashan Hospital affiliated to Fudan University (KY2018‐250:01) and complies with the Declaration of Helsinki and its subsequent revisions.

### 2.2 Animals

This study used male 5xFAD mice on a C57BL/6 background as the AD model because they provide a robust and reproducible amyloid‐associated neuroinflammatory background for examining whether long‐term HFD‐induced metabolic stress further exacerbates AD‐like neuroimmune dysregulation and Treg dysfunction.^29^ Additionally, littermate wild‐type (WT) mice were used as the control group. Mice were purchased from Cavens Laboratory Animal Co., Ltd (Changzhou, China). All mice were housed in an environment of 20°C to 22°C with a 12‐hour light/dark cycle until 8 to 10 weeks of age. WT and 5xFAD mice were randomly divided into control diet (CD) group and HFD group, with 15 mice in each group: WT‐CD, WT‐HFD, AD‐CD, and AD‐HFD (CD mice were fed a standard diet containing 5% to 10% fat, while HFD mice were fed a high‐fat diet containing 60% fat; the feeding period was 20 weeks). During the period, all the mice were weighed, and their health status and signs were observed weekly. At the end of the feeding period, mice underwent blood glucose measurement, behavioral testing, and neuroimaging. Before sample collection, mice were deeply anesthetized with sodium pentobarbital. All procedures were performed in accordance with the National Institutes of Health Guidelines for the Care and Use of Laboratory Animals and approved by the Department of Laboratory Animal Science, Fudan University (Approval No. 202407015S).

**RESEARCH IN CONTEXT**

1. **Systematic review**: Metabolic disorders, including obesity and type 2 diabetes, are associated with increased risk of cognitive decline and Alzheimer's disease (AD). Prior studies have linked high‐fat diet and glucolipotoxic stress to amyloid pathology, neuroinflammation, and synaptic dysfunction, but how metabolic stress disrupts regulatory T cell (Treg)‐mediated neuroimmune regulation remains unclear.
2. **Interpretation**: Metabolic stress induces Z‐DNA‐binding protein 1 (ZBP1) upregulation in Tregs, leading to impaired Treg stability, apoptosis/necroptosis‐related signaling, and reduced suppressive function via the T‐cell receptor (TCR) signal. ZBP1‐deficient Tregs better restrained microglial activation, astrocytic C3/A1‐like responses, neuronal injury, amyloid‐associated pathology, metabolic impairment, synaptic dysfunction, and cognitive decline.
3. **Future directions**: Future work would examine whether ZBP1‐associated Treg dysfunction occurs in patients with AD and metabolic disorders, clarify how ZBP1 alters proximal TCR signaling, and test whether preserving Treg fitness can reduce metabolically driven neuroimmune injury.

### 2.3 Intraperitoneal glucose tolerance test and insulin tolerance test

Glucose levels in mice were assessed using a Roche blood glucose meter and type C test strips (Roche). Both the intraperitoneal glucose tolerance test (IPGTT) and insulin tolerance test (ITT) were performed at the end of the feeding period. In the IPGTT, mice were fasted for 14 hours and then injected with 2 g/kg of 40% glucose solution. Blood glucose levels were measured at 0, 30, 60, and 120 minutes post‐injection to calculate the IPGTT value. In the ITT, mice were fasted overnight and then injected with 0.75 IU/kg of insulin. Plasma glucose levels were measured at the same time points as in the IPGTT.

### 2.4 Behavioral assessments

#### 2.4.1 Morris water maze test

The Morris water maze (MWM) test was performed as previously described.^30^ Briefly, the MWM test assesses the spatial learning and memory abilities of mice by placing them in a circular pool filled with opaque, temperature‐controlled water. Mice are trained for 5 days to use spatial cues to find underwater platforms. On the test day, the platforms are removed, and the mice's swimming trajectory is tracked using EthoVision XT 8.5 (Noldus).

#### 2.4.2 Y‐maze test

The Y‐maze test was used to assess spontaneous alternation behavior and working memory in laboratory animals. Mice were placed at the intersections of a Y‐maze and allowed to explore freely for 8 minutes. Spontaneous alternation behavior was measured by the number of consecutive entries into all three arms. Working memory was calculated as the ratio of actual alternations to the maximum possible alternations. Motion and data analysis were performed using EthoVision XT14 software (Noldus).

### 2.5 Magnetic resonance imaging

T2‐weighted imaging scans were performed using an 11.7T magnetic resonance imaging (MRI) system (Bruker Biospin). Mice were anesthetized with 2.5% isoflurane and placed in an MRI coil for scanning, maintained using 1% isoflurane. Body temperature was maintained using a warming blanket, and heart rate and respiratory rate were monitored in real time. T2WI scan parameters were set as follows: repetition time (TR)/echo time (TE) = 3000/26 ms, field of view (FOV) = 18×18 mm, acquisition matrix = 256×256, 45 slices, slice thickness 0.3 mm, averaged twice, and rapid relaxation enhancement acquisition (RARE) factor of 8.

### 2.6 Thioflavine S staining

Thioflavin S staining was used to detect amyloid plaques in mouse brain sections. Sections were incubated with freshly prepared Thioflavin S (T1892, Sigma Aldrich) solution for 8 minutes at room temperature in the dark. After staining, sections were briefly differentiated twice in 80% ethanol for 10 to 20 seconds, rinsed four to five times in ddH_2_O, and incubated in high‐concentration phosphate buffer solution for 30 minutes in the dark. Sections were then washed three to five times with double‐distilled water and mounted with antifade medium containing 4′,6‐diamidino‐2‐phenylindole (DAPI).

### 2.7 Immunofluorescence staining and image analysis

Frozen coronal brain sections (25 µm) were subjected to immunofluorescence staining using standard procedures. Sections were blocked in 5% donkey serum and incubated overnight at 4°C with primary antibodies diluted in phosphate‐buffered saline (PBS) supplemented with 0.3% triton x‐100 and 1% goat or donkey serum. After thorough washes, sections were incubated with species‐appropriate fluorescent secondary antibodies for 2 hours at room temperature. Antibody details are listed in Table S1 in supporting information. Nuclei were counterstained, and sections were mounted with DAPI fluoromount‐G (36308ES20, Yesen). Images were captured using a Nikon AX confocal microscope (AX Ti2E, Nikon) and analyzed with ImageJ 1.54r (National Institutes of Health) and Imaris 10.0.0 (Oxford Instruments).

### 2.8 Western blot analysis

Total proteins were extracted from tissues and cultured cells using radioimmunoprecipitation assay lysis buffer. Protein concentrations were measured using a bicinchoninic acid protein assay kit (Beyotime). Equal amounts of protein were denatured in loading buffer, resolved by sodium dodecyl sulfate‐polyacrylamide gel electrophoresis (SDS‐PAGE), and transferred onto polyvinylidene fluoride membranes (Millipore). Membranes were then blocked and incubated overnight at 4°C with the indicated primary antibodies. After Tris‐buffered saline polysorbate 20 washes, membranes were incubated with horseradish peroxidase–conjugated secondary antibodies for 2 hours at room temperature. Antibody details are listed in Table S1. Protein bands were detected using an enhanced chemiluminescence kit (Millipore) and visualized with a chemiluminescence imaging system. Band intensity was quantified using ImageJ 1.54r.

### 2.9 Transcriptome sequencing

Total RNA was extracted from hippocampal tissues of the AD‐CD group (*n =* 4) and AD‐HFD group (*n =* 5) using TRIzol reagent (Invitrogen), followed by DNase I treatment (Takara) to remove genomic DNA. After RNA purification and library preparation, paired‐end sequencing was performed on the DNBSEQ‐T7 platform (MGI Tech Co., Ltd.) in PE150 mode. Raw reads were processed with Trimmomatic (v0.36) to remove adaptors and low‐quality reads. Clean reads were aligned to the reference genome using STAR (v2.5.3a), and gene‐level read counts were quantified using featureCounts (Subread v1.5.1). Differential expression analysis between the AD‐HFD and AD‐CD groups was performed using the edgeR package in R based on raw count data. Genes with *p* < 0.05 were considered differentially expressed. Differentially expressed genes were visualized using a volcano plot based on log2 fold change and −log10 (*p* value). To characterize the biological processes associated with transcriptional alterations, Gene Ontology (GO) enrichment analysis was performed separately for upregulated and downregulated differentially expressed genes in R. Significantly enriched GO biological process (BP) terms were visualized as bar plots according to gene counts.

### 2.10 Flow cytometry analysis

Brain tissues were mechanically dissociated and enzymatically digested using a trypsin‐based dissociation solution at 37°C for 30 minutes, followed by gentle mechanical dissociation. The resulting cell suspension was filtered through a 70 µm cell strainer and washed with Hank's Balanced Salt Solution (HBSS). Mononuclear cells were isolated by Percoll density gradient centrifugation (800 g, 30 minutes, 18°C), and cells at the interphase were collected, washed, and resuspended in HBSS containing 2% fetal bovine saline (FBS). Cells were incubated with anti‐CD16/CD32 antibodies to block Fc receptor‐mediated non‐specific binding for 10 minutes at 4°C. Surface staining was then performed using fluorophore‐conjugated antibodies for 30 minutes at 4°C in the dark. For intracellular Foxp3 staining, cells were fixed and permeabilized using a permeabilization kit (Thermo Fisher) according to the manufacturer's instructions, then incubated with an anti‐Foxp3 antibody.

Peripheral blood samples were treated with ammonium–chloride–potassium lysis buffer to remove erythrocytes and processed using the same staining protocol. For flow cytometric analysis, debris and doublets were excluded based on forward scatter cytometry (FSC)/side scatter cytometry (SSC) parameters and FSC area versus FSC height gating, respectively. Live cells were identified by exclusion of viability dye‐positive cells. CD3^+^ T cells were gated, followed by selection of CD4^+^ T cells. Tregs were defined as CD4^+^ CD25^+^ Foxp3^+^ cells (Figure S1 in supporting information). Data were acquired on a CytoFLEX flow cytometer (Beckman Coulter) and analyzed using FlowJo V10.10 (FlowJo LLC). Fluorophores for flow cytometry are provided in Table S2 in supporting information.

### 2.11 Splenic Tregs enrichment and validation

Spleens from WT‐CD, WT‐HFD, AD‐CD, and AD‐HFD mice were mechanically dissociated and filtered through a 70 µm cell strainer to generate single‐cell suspensions, which were subsequently washed with HBSS supplemented with 5% FBS. Cells were counted and adjusted to a final concentration of 1 × 10^8^ cells mL^−^^1^. Splenic CD4^+^ T cells were first isolated using the EasySep Mouse CD4^+^ T Cell Isolation Kit (STEMCELL Technologies). Tregs were then enriched from the CD4^+^ T cell fraction using the EasySep Mouse CD25 Regulatory T Cell Positive Selection Kit (STEMCELL Technologies) according to the manufacturer's instructions. The remaining non‐Treg CD4^+^ CD25^−^ population was used as conventional T cells (Tconvs) where indicated. The enrichment efficiency of splenic Tregs was assessed by flow cytometry before and after magnetic isolation. Cells were stained for CD25 and intracellular Foxp3, and enriched Tregs were identified as CD25^+^Foxp3^+^ cells. Enriched Tregs were subsequently used for western blot analysis.

### 2.12 Primary T cell culture and treatment

Primary splenic CD4^+^ T cells, Tregs, and Tconvs were isolated from WT and ZBP1‐KO (knockout) mice as described above. For T cell activation, six‐well plates were coated overnight at 4°C with Ultra‐LEAF Purified anti‐mouse CD3ε antibody (5 µg mL^−^^1^; 100340, BioLegend), washed three times with PBS, and seeded with purified cells at 1 × 10^6^ cells per well in complete culture medium. Ultra‐LEAF Purified anti‐mouse CD28 antibody (2 µg mL^−^^1^; 102121, BioLegend) was added as a soluble co‐stimulatory signal. Cells were maintained at 37°C in a humidified incubator with 5% CO_2_. For Treg culture, recombinant mouse interleukin (IL)‐2 (20 ng mL^−^^1^; 575406, BioLegend) and recombinant mouse transforming growth factor beta 1 (TGF‐β1; 5 ng mL^−^^1^; HY‐P7117, MedChemExpress) were added to maintain Treg phenotype and function. When indicated, cells were subjected to the corresponding treatments and collected for subsequent analyzes. Tregs were treated with high glucose and high palmitate (HGHP; 33 mM glucose and 300 µM palmitic acid) for 48 hours. Palmitic acid was conjugated to fatty acid–free bovine serum albumin before use.

### 2.13 Carboxyfluorescein succinimidyl ester–based suppression assay

Responder Tconvs were labelled with carboxyfluorescein succinimidyl ester (CFSE) using a CFSE Cell Division Tracker Kit (423801, BioLegend). Briefly, cells were washed with PBS, resuspended at 1×10^7^ cells mL^−^^1^, and incubated with 2.5 µM CFSE for 5 minutes at room temperature. Staining was quenched by the addition of an equal volume of complete medium containing 10% FBS for 10 minutes, followed by washing to remove excess dye. CFSE‐labelled Tconvs were then cultured alone or co‐cultured with Tregs at a 1:1 ratio under anti‐CD3/CD28 stimulation. Tregs from WT or ZBP1‐KO mice, with or without HGHP treatment, were used as indicated. After 96 hours, Tconv proliferation was quantified by flow cytometry based on CFSE dilution. Treg‐mediated suppression was calculated as follows: suppression (%) = (1 – [proliferation rate of Tconv cells co‐cultured with Tregs / proliferation rate of stimulated Tconv cells cultured alone]) × 100.

### 2.14 Immunoprecipitation

Tregs were lysed in lysis/wash buffer using an Immunoprecipitation Kit with Protein A+G Magnetic Beads (Beyotime), and lysates were clarified by centrifugation at10700 ×g for 5 to 10 minutes at 4°C. The supernatants were collected for immunoprecipitation. For each sample, 500 µg of total protein was incubated with 5 µg of primary antibody overnight at 4°C in a final volume of 500 µL to allow formation of antigen–antibody complexes. Reciprocal immunoprecipitation was performed using anti‐LCK antibody or anti‐ZBP1 antibody, as indicated. Protein A+G magnetic beads were washed twice with lysis/wash buffer and incubated with the immune complexes for 30 minutes at room temperature with rotation. Beads were collected using a magnetic rack and washed three times with lysis/wash buffer. Bound proteins were eluted in 1×SDS‐PAGE loading buffer, denatured at 95°C for 5 minutes, and analyzed by immunoblotting. Antibody details are listed in Table S1.

### 2.15 Molecular dynamics simulation and binding interface analysis

Molecular dynamics (MD) simulations were performed to evaluate the structural stability and binding interface of the ZBP1‐LCK complex. The initial complex structure was obtained from molecular docking, and the top‐ranked conformation was selected for simulation. Simulations were carried out using GROMACS 2025.1 with the Amber14SB force field. The complex was solvated in a cubic water box, energy minimized, and equilibrated under canonical (NVT) and microcanonical (NPT) ensembles. Production simulation was performed for 100 ns at 300K and 1 bar with a 2fs time step. Long‐range electrostatic interactions were treated using the particle mesh Ewald (PME) method, and bonds involving hydrogen atoms were constrained using the LINCS algorithm. Trajectory frames were saved every 30 ps for subsequent analysis. After alignment to the protein backbone, root mean square deviation was calculated to assess structural stability. Representative conformations from the equilibrated phase were used for interface analysis. Residue–residue contact patterns of ZBP1, LCK, and the ZBP1–LCK complex were visualized as contact maps, and key interacting residues were further displayed in the three‐dimensional structural model of the complex.

### 2.16 Primary glial cell isolation and culture

Primary glial cultures were prepared from the hippocampi of postnatal day 1 to 3 (P1–P3) neonatal mice. Briefly, brains were rapidly dissected in ice‐cold Dulbecco's Modified Eagle Medium (DMEM)/F12 medium prepared from DMEM, high glucose (11965118, Gibco) and Ham's F‐12 Nutrient Mixture (11765062, Gibco), and the meninges were carefully removed under a stereomicroscope. The hippocampi were isolated, minced, and enzymatically digested with papain, followed by gentle trituration and filtration through a 40 µm cell strainer. The resulting mixed glial cells were collected by centrifugation and seeded into poly‐D‐lysine‐coated T75 flasks (poly‐D‐lysine, A3890401, Gibco) in serum‐containing DMEM/F12 medium supplemented with penicillin‐streptomycin (15140148, Gibco). After 24 hours, the medium was completely replaced, and cultures were maintained for 9 to 10 days. Primary microglia were obtained by shaking mixed glial cultures at for 2 hours at 37°C. Detached microglia were collected and replated for subsequent experiments, while the remaining adherent cells were maintained as astrocyte‐enriched cultures for downstream analyses.

### 2.17 Primary microglia–Treg transwell co‐culture and treatment

Primary microglia were seeded into the lower chamber of transwell inserts (0.4 µm pore size), while purified Tregs were placed in the upper chamber to establish a non‐contact co‐culture system. Tregs were isolated from WT or ZBP1‐KO mice as indicated and maintained in RPMI 1640 medium supplemented with penicillin–streptomycin. Primary microglia in the lower chamber were treated with Aβ_1‐42_ (3 µM; 20602ES03, Yeasen) for 48 hours. During the entire co‐culture period, Tregs in the upper chamber were maintained under the indicated conditions, with or without HGHP treatment. After treatment, microglia were collected for downstream analyses, including immunofluorescence staining and immunoblotting.

### 2.18 Latex bead phagocytosis assay

To evaluate microglial phagocytic activity, primary microglia in the lower chamber were stimulated with interferon gamma (IFN‐γ; 20 ng/mL; I4777, Sigma‐Aldrich) and lipopolysaccharide (LPS; 100 ng/mL; HY‐D1056, MedChemExpress) for 24 hours under Transwell co‐culture conditions. In parallel, Tregs in the upper chamber were maintained with or without HGHP treatment, as indicated. After inflammatory stimulation, red fluorescent amine‐modified polystyrene latex beads (L2778, Sigma‐Aldrich) were added to the lower chamber and incubated for 4 hours to allow phagocytosis. Cells were then washed extensively with PBS to remove non‐internalized beads and harvested for flow cytometric analysis. Phagocytic activity was quantified as the percentage of bead‐positive microglia.

### 2.19 Primary astrocyte–Treg co‐culture and treatment

Astrocyte‐enriched cultures remaining adherent in the flasks were further maintained in DMEM (11965118, Gibco) supplemented with penicillin–streptomycin and used for subsequent co‐culture experiments. For non‐contact co‐culture, astrocytes were seeded into the lower chamber of transwell plates, and purified Tregs from WT or ZBP1‐KO mice were placed in the upper chamber. For stimulation, astrocytes were exposed to Aβ_1‐42_ (3 µM) for 48 hours, while upper‐chamber Tregs were maintained with or without HGHP treatment throughout the co‐culture period. After treatment, astrocytes were collected for immunofluorescence staining, mitochondrial membrane potential analysis, and immunoblotting, as indicated.

### 2.20 Primary neuronal isolation and culture

Primary hippocampal neurons were prepared from embryonic day 16. Briefly, hippocampi were dissected under a stereomicroscope, dissociated by papain digestion, and filtered through a 40 µm cell strainer to obtain a single‐cell suspension. To enrich neurons, dissociated cells were subjected to Percoll density gradient centrifugation, and cells at the interphase were collected, washed, and resuspended in culture medium. Cell viability was assessed by trypan blue exclusion. Culture plates were pre‐coated with poly‐D‐lysine (A3890401, Gibco). Neurons were initially seeded in serum‐containing DMEM, high glucose (cat. no. 11965118, Gibco) at a density of 1×10^6^ cells per well in six‐well plates. After 1 hour, the medium was replaced with Neurobasal medium (21103049, Gibco) supplemented with B‐27 Plus Supplement (A3582801, Gibco), GlutaMAX Supplement (35050‐061, Gibco), and penicillin–streptomycin (15140148, Gibco). Medium was changed again after 24 hours, and neurons were cultured for 7 to 10 days before subsequent experiments.

### 2.21 Primary neuron–Treg co‐culture and treatment

Primary hippocampal neurons were used for co‐culture experiments after 7 to 10 days in vitro. For non‐contact co‐culture, neurons were seeded in the lower chamber of transwell plates, and purified Tregs isolated from WT or ZBP1‐KO mice were placed in the upper chamber. Tregs were maintained under the indicated conditions, with or without HGHP treatment. For neuronal injury experiments, neurons in the lower chamber were exposed to Aβ_1‐42_ (3 µM) for 48 hours, while upper‐chamber Tregs were maintained throughout the co‐culture period. After treatment, neurons were collected for immunofluorescence staining, immunoblotting, or mitochondrial membrane potential analysis.

### 2.22 JC‐1 staining

Mitochondrial membrane potential was assessed using a JC‐1 mitochondrial membrane potential assay kit (C2006, Beyotime). After the indicated treatments, neurons were incubated with JC‐1 working solution at 37°C for 20 minutes in the dark, followed by washing with the provided buffer according to the manufacturer's instructions. Cells were immediately imaged using a fluorescence microscope. JC‐1 aggregates in polarized mitochondria emitted red fluorescence, whereas JC‐1 monomers in depolarized mitochondria emitted green fluorescence. Mitochondrial membrane potential was quantified as the ratio of red to green fluorescence intensity.

### 2.23 Positron emission tomography–computed tomography imaging

Positron emission tomography (PET) computed tomography (CT) imaging was performed at Huashan Hospital (Shanghai, China). Mice were fasted overnight and anesthetized with 2.5% isoflurane for induction and 1.0% isoflurane for maintenance during the entire imaging session. At 30 minutes after tail‐vein administration of F^18^ fluorodeoxyglucose (F^18^‐FDG) or F^18^ florbetapir (F^18^‐AV45; 200–250 µCi), dynamic PET data were acquired for 20 minutes. Image processing was conducted using an established mouse brain PET analysis pipeline incorporating predefined volume of interest (VOI) templates. The hippocampus was selected as the VOI for quantitative analysis. Template VOIs were spatially normalized to upright CT images, which had been reoriented to a standardized vertical midline position, using skull‐based CT to standard CT normalization parameters generated in Statistical Parametric Mapping (SPM). PET images were then co‐registered to the corresponding CT images with the SPM5 registration module using a mutual information algorithm. Standardized uptake value ratios (SUVRs) were calculated for each time point using the cerebellum as the reference region.

### 2.24 Field potential recordings

Acute hippocampal slices were prepared as described.^31^ Field excitatory postsynaptic potentials (fEPSPs) were recorded from the CA1 stratum radiatum after stimulation of Schaffer collateral fibers. Synaptic responses were evoked by biphasic rectangular current pulses (100 µs per polarity) and amplified using an EXT‐20F differential amplifier (npi electronic GmbH) with a 3 kHz low‐pass filter and 0.1 Hz high‐pass filter. Data were digitized at 10 kHz using a CED 1401 plus AD/DA converter (Cambridge Electronic Design). The initial slope of the fEPSP was used as a measure of synaptic strength. Stimulation intensity was set to 40% to 50% of the maximal response, and a stable baseline was established before induction. Theta burst stimulation was used to induce synaptic potentiation. fEPSP slopes were normalized to the mean baseline value, and synaptic potentiation was assessed as the percentage of baseline at 120 minutes after Theta burst stimulation.

### 2.25 Statistical analysis

Statistical analyses were performed using GraphPad Prism 10 (GraphPad Software). Sample sizes were selected based on previous studies using comparable experimental models and the availability of biological samples.^30^ The animal or independently prepared cell culture was considered the biological replicate, and technical replicates were averaged before statistical analysis. Sample sizes are specified in the corresponding figure legends. Within each genotype or experimental condition, mice were randomly assigned to diet or treatment groups using a random‐number generator. Investigators performing behavioral testing, imaging, sample processing, data acquisition, and quantitative analysis were blinded to group allocation until completion of the analyses. Data are presented as mean ± standard deviation (SD). Normality was assessed using the Shapiro–Wilk test. Comparisons between two groups were performed using an unpaired two‐tailed Student *t* test or Welch *t* test, as appropriate. Comparisons among multiple groups were performed using one‐way or two‐way analysis of variance (ANOVA) followed by a Tukey multiple‐comparisons test, or a Welch ANOVA when variances were unequal. For transcriptomic analyses, multiple testing was controlled using the Benjamini–Hochberg false discovery rate procedure. All tests were two sided, and adjusted *p* < 0.05 was considered statistically significant.

## 3 RESULTS

### 3.1 Metabolic stress is associated with aggravated cognitive impairment in individuals with T2DM and HFD‐fed 5xFAD mice

To explore whether metabolic disturbance is associated with more severe cognitive dysfunction, we conducted community‐based cognitive screening using the MMSE and identified 290 individuals with cognitive impairment (Figure 1A), including 102 with T2DM and 188 without T2DM (Table S3 in supporting information). Compared to non‐T2DM participants, those with T2DM were older and had different education distributions, while sex, height, weight, waist circumference, and body mass index (BMI) were comparable between groups (Table S3). Among cognitively impaired individuals, T2DM participants exhibited more severe cognitive impairment (Figure 1B), with significantly lower MMSE scores (21 [14–24] vs. 23 [20–24], *p* < 0.001; Figure 1C). Moreover, SCD‐Q9 scores were higher in the T2DM group (5.5 [3–7.5] vs. 4.5 [2–6.5], *p* < 0.001; Figure 1D). Given that age, sex, education attainment, and vascular–metabolic comorbidities may influence cognitive performance, we further performed multivariable regression analyses to account for potential confounding. After adjustment for age, sex, education, BMI, hypertension, and hyperlipidemia, T2DM remained associated with lower MMSE scores and higher SCD‐Q9 scores (Tables S4 and S5 in supporting information). These findings support an association between T2DM‐related metabolic abnormality and aggravated cognitive impairment in humans.

**FIGURE 1:**
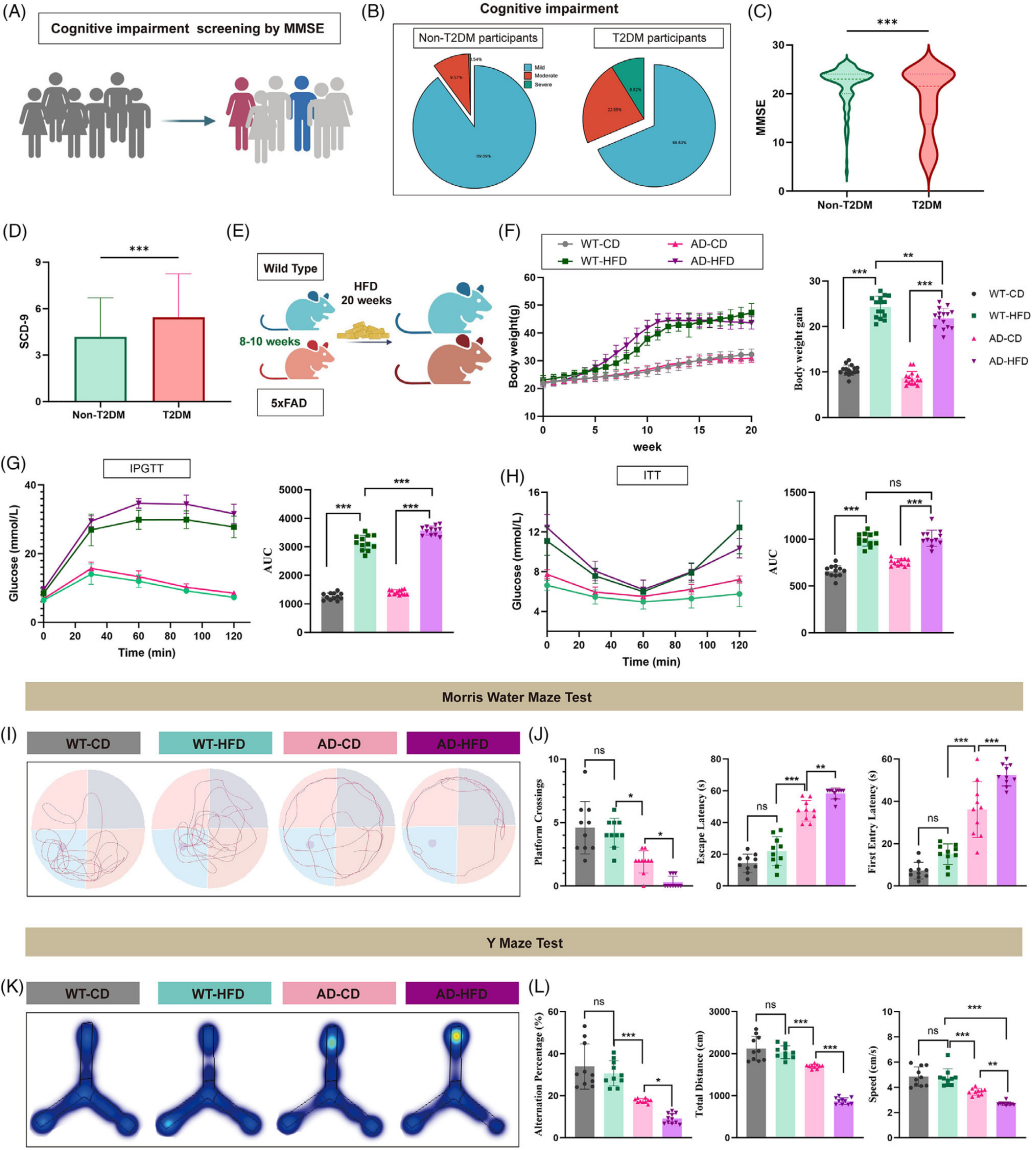
Metabolic dysfunction is associated with aggravated cognitive impairment in individuals with T2DM and HFD‐fed 5xFAD mice. A, Schematic overview of community‐based cognitive screening using the MMSE. B, Distribution of cognitive impairment severity among cognitively impaired older adults with or without T2DM. C, D, Comparison of MMSE scores (C) and SCD‐Q9 scores (D) between non‐T2DM and T2DM participants. E, Animal experimental design, WT and 5xFAD mice fed a CD or a HFD for 20 weeks (*n =* 12 per group). F, Longitudinal body weight changes and total body weight gain in mice fed CD or HFD. G, Quantitative analysis of the IPGTT and its AUC in each group of mice; and (H) quantitative analysis of the AUC of the ITT. I, J, The MWM test was used to assess spatial memory. I, A representative swimming path is shown in the figure; (J) quantitative analysis of platform crossings, escape latency, and first entry latency (*n =* 10 per group). K, L, The Y‐maze test was used to assess spatial working memory. K, A representative movement trajectory heatmap is shown in the figure; (L) quantitative analysis of the percentage of spontaneous alternations, total distance, and speed (*n *= 10 per group). Data are expressed as mean ± standard deviation; *p < 0.05, **p < 0.01, ***p < 0.001. AD, Alzheimer's disease; AUC, area under the curve; CD, control diet; HFD, high‐fat diet; IPGTT, intraperitoneal glucose tolerance test; ITT, insulin tolerance test; MMSE, Mini‐Mental State Examination; MWM, Morris water maze; SCD‐Q9, 9‐item Subjective Cognitive Decline Questionnaire; T2DM, type 2 diabetes mellitus; WT, wild type.

Based on the clinical association between T2DM and poorer cognitive performance, we next used HFD‐fed 5xFAD mice to investigate whether chronic metabolic stress could exacerbate amyloid‐associated neuroimmune dysfunction in an AD‐like pathological background. We used 5xFAD mice fed either a CD or a HFD for 20 weeks, with WT littermates included as controls (Figure 1E). HFD feeding markedly increased body weight gain in both WT and 5xFAD mice (Figure 1F). HFD‐fed mice displayed impaired glucose tolerance and reduced insulin sensitivity, as evidenced by increased glucose excursion and area under the curve (AUC) in the IPGTT and ITT, respectively (Figure 1G and 1H). In the MWM test, HFD‐fed 5xFAD mice showed marked impairments in spatial learning and memory, as reflected by fewer platform crossings, prolonged latency to first enter the hidden platform, and target quadrant (Figure 1I and 1J). In line with these findings, both IPGTT‐AUC and ITT‐AUC were positively correlated with escape latency and first‐entry latency, but negatively correlated with platform crossings (Figure S2 in supporting information). In parallel, HFD‐fed 5xFAD mice displayed reduced spontaneous alternation, locomotor activity, and movement speed in the Y‐maze test (Figure 1K and 1L). Likewise, higher IPGTT‐AUC and ITT‐AUC values were associated with lower spontaneous alternation and reduced movement speed in the Y‐maze test (Figure S3 in supporting information). Together, these findings suggest that 5xFAD mice are more susceptible to HFD‐induced cognitive and behavioral impairment than WT mice, supporting a close relationship between systemic metabolic disturbance and behavioral impairment.

### 3.2 HFD‐induced metabolic stress aggravates hippocampal amyloid pathology, neurodegeneration, and gliosis in 5xFAD mice

We further investigated whether HFD exacerbated hippocampal pathology in 5xFAD mice. MRI analysis revealed that hippocampal volume and hippocampal fraction were reduced in 5xFAD mice relative to WT controls and were further decreased by HFD feeding (Figure 2A–C), indicating aggravated hippocampal atrophy under metabolic challenge. Consistently, Thioflavin S staining showed increased amyloid plaque burden in the CA1, CA3, and dentate gyrus regions of HFD‐fed 5xFAD mice compared to control diet–fed 5xFAD mice (Figure 2D–G). At the molecular level, HFD‐fed 5xFAD mice exhibited increased expression of beta‐secretase 1, neurofilament light chain, and glial fibrillary acidic protein (GFAP), together with reduced postsynaptic density protein 95 levels (Figure 2H and 2I), supporting enhanced amyloidogenic processing, neuronal injury, astrogliosis, and synaptic damage. These observations were further supported by increased hippocampal ionized calcium‐binding adapter molecule 1 and GFAP immunoreactivity, indicating enhanced microglial and astrocytic activation in HFD‐fed 5xFAD mice (Figure S4A–D in supporting information). Golgi staining showed that dendritic spine density was reduced in 5xFAD mice and further decreased by HFD feeding, with concomitant reduction in mushroom spines and an increase in thin and stubby spines (Figure S4E–G).

**FIGURE 2:**
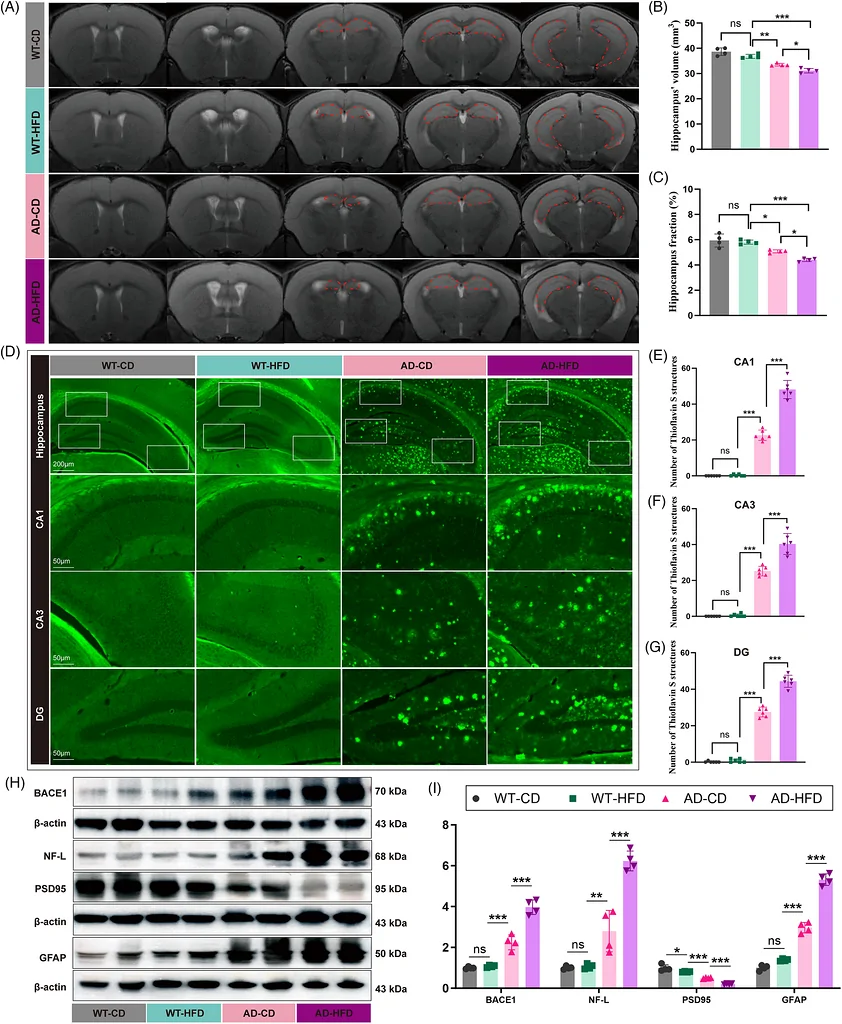
HFD feeding aggravates hippocampal atrophy, amyloid deposition, and neurodegenerative pathology in AD mice. A, Representative T2‐weighted MRI images from WT‐CD, WT‐HFD, AD‐CD, and AD‐HFD mice. Red dashed lines indicate hippocampal boundaries. B, C, Quantification of hippocampal volume (B) and hippocampal fraction (C) in the indicated groups (*n =* 4 per group). D, Representative Thioflavin S staining of hippocampal sections showing amyloid plaque burden in the whole hippocampus and in the CA1, CA3, and DG subregions. Scale bars, 200 µm. Boxed areas are shown at higher magnification. Scale bars, 50 µm. E‐G, Quantification of Thioflavin S–positive plaque number in CA1, CA3, and DG (*n =* 6 per group). H, Representative immunoblots of BACE1, NfL, PSD95, and GFAP in hippocampal tissue from the indicated groups. β‐actin serves as a loading control. I, Densitometric quantification of BACE1, NfL, PSD95, and GFAP normalized to β‐actin (*n =* 4 per group). Data are presented as mean ± standard deviation; NS, not significant; *P < 0.05, **P < 0.01, ***P < 0.001. AD, Alzheimer's disease; BACE1, beta‐secretase 1; CD, control diet; DG, dentate gyrus; GFAP, glial fibrillary acidic protein; HFD, high‐fat diet; MRI, magnetic resonance imaging; NfL, neurofilament light chain; PSD95, postsynaptic density protein 95; WT, wild type.

### 3.3 HFD‐induced metabolic stress activates ZBP1‐related programmed cell death in Tregs of 5xFAD mice

We investigated the transcriptomic profiles of hippocampal tissues from AD‐HFD and AD‐CD mice, revealing that there was a significant upregulation of ZBP1 expression in the AD‐HFD group (Figure 3A). Functional categorization of differentially expressed genes further showed that HFD‐responsive transcriptional changes were enriched in immune‐related processes, including cell activation, cytokine regulation, antigen processing and presentation, and adaptive immune responses (Figure 3B). These findings suggested that HFD induces marked immune remodeling in 5xFAD mice. To define the immune cell context of ZBP1 expression, we interrogated publicly available single‐cell and immune‐cell expression datasets. These analyses showed that ZBP1 was expressed across several immune populations, with relatively high expression in T‐cell and Treg subsets (Figure S5A–D in supporting information). Given the central role of Tregs in immune regulation, we investigated whether the increase in ZBP1 was associated with alterations in the Treg population. Flow cytometry analysis showed that decreased proportion of Tregs in both brain tissue and peripheral blood of 5xFAD mice, with HFD further reducing the proportion of Tregs, while no significant difference was observed between WT‐CD and WT‐HFD mice (Figure 3C–E). These results suggest that a HFD exacerbates Treg loss in AD. Immunofluorescence staining showed that ZBP1 co‐localized with Foxp3‐positive Tregs (Figure 3F). To further validate this observation, we isolated CD4^+^CD25^+^ Tregs from mouse spleens and assessed the enrichment efficiency by flow cytometry. The proportion of CD25^+^Foxp3^+^ cells increased from 2.65% in the unsorted population to 87.4% after enrichment, confirming efficient Treg enrichment (Figure 3G). Quantitative polymerase chain reaction (PCR) analysis of purified Tregs showed that Zbp1 mRNA expression was significantly increased in AD‐HFD mice compared to AD‐CD mice, whereas no significant difference was observed between the WT‐CD and WT‐HFD groups (Figure S6 in supporting information). Immunoblot analysis of purified Tregs showed significantly increased ZBP1 expression, along with elevated levels of gasdermin D and phosphorylated mixed lineage kinase domain‐like (MLKL) in the AD‐HFD mice, compared to the AD‐CD group (Figure 3H–K). These findings suggest that HFD‐induced metabolic stress engages ZBP1‐associated programmed cell death in Tregs and identify Treg‐associated ZBP1 activation as a prominent immunological feature of HFD‐exacerbated AD pathology.

**FIGURE 3:**
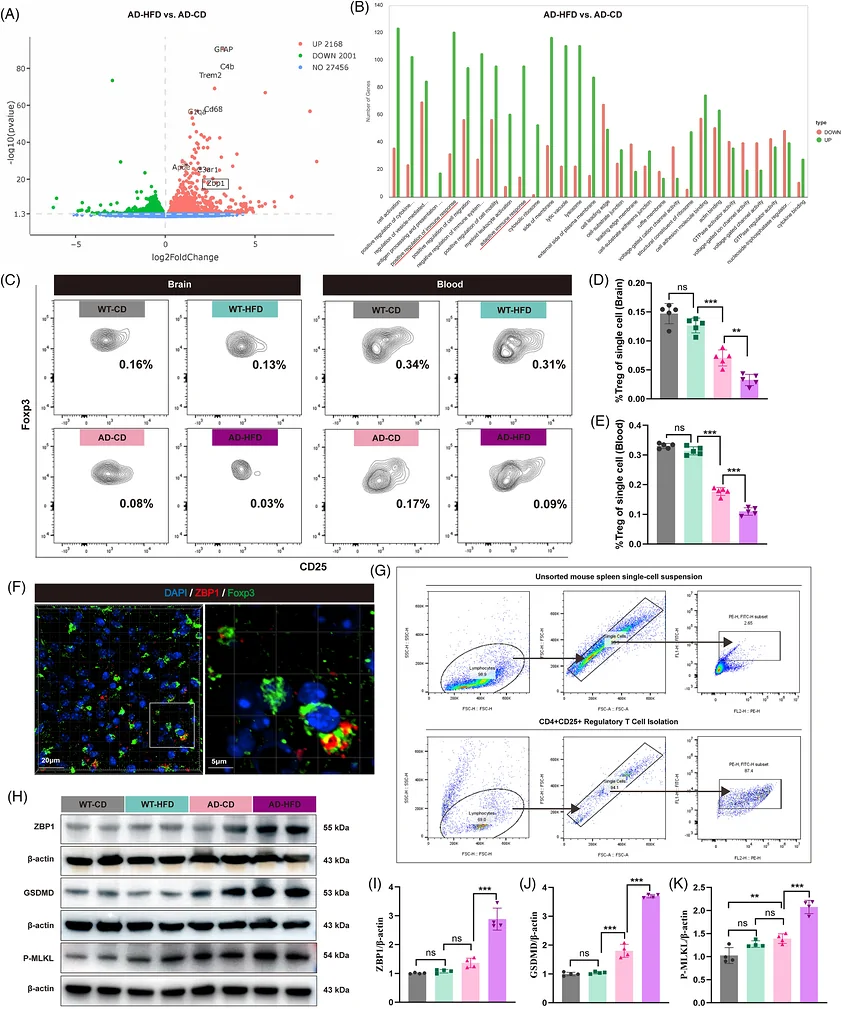
HFD feeding induces ZBP1‐associated Treg depletion and programmed cell death signaling in AD mice. A, Volcano plot showing differentially expressed genes in hippocampal tissue from AD‐HFD versus AD‐CD mice. B, Gene Ontology biological process enrichment analysis of differentially expressed genes. C, Representative flow‐cytometric plots of Treg cells in brain tissue and peripheral blood from WT‐CD, WT‐HFD, AD‐CD, and AD‐HFD mice. Quantification of Treg frequency among single cells in brain (D) and blood (E; *n =* 5 per group). F, Representative immunofluorescence images showing ZBP1 expression in Foxp3^+^ Treg cells in brain tissue. Nuclei are counterstained with DAPI. Scale bars, 20 µm (left) and 5 µm (right). G, Representative flow cytometry plots showing Treg% from the mouse spleen. CD4^+^CD25^+^ Tregs accounted for 2.65% of unsorted mouse spleen single cells and 87.4% of sorted cells after enrichment. The enriched Treg fraction was used for immunoblot analysis in (H)–(K). H, Representative immunoblots of ZBP1, GSDMD, and p‐MLKL in splenic Treg cells from the indicated groups. I–K, Densitometric quantification of ZBP1, GSDMD, and p‐MLKL normalized to β‐actin (*n =* 4 per group). Data are presented as mean ± standard deviation; NS, not significant; *P < 0.05, **P < 0.01, ***P < 0.001. AD, Alzheimer's disease; CD, control diet; DAPI, 4′,6‐diamidino‐2‐phenylindole; GSDMD, gasdermin D; HFD, high‐fat diet; MLKL, mixed lineage kinase domain‐like; Treg, regulatory T cell; WT, wild type; ZBP1, Z‐DNA‐binding protein 1.

### 3.4 ZBP1 deficiency attenuates HGHP‐induced necroptotic and apoptotic signaling in Tregs

To investigate the role of ZBP1 in Treg stability under HGHP conditions, primary CD4^+^T cells were isolated from WT and ZBP1‐KO mice. The genotype of ZBP1‐KO mice was confirmed by PCR‐based genotyping before cell isolation (Figure S7 in supporting information). CD4^+^T cells were activated with CD3 and CD28 antibodies, cultured under IL‐2 and TGF‐β to induce Treg differentiation (Figure 4A, B). Flow cytometry showed that HGHP treatment reduced the percentage of Tregs in the WT group, while the ZBP1‐KO group maintained a higher proportion of Tregs, even after HGHP treatment, compared to WT‐HGHP (Figure 4C–D). Apoptosis analysis revealed that HGHP treatment significantly increased the total apoptosis rate in WT Tregs (Figure 4E, F). However, ZBP1‐KO‐HGHP Tregs showed a significant reduction in apoptosis compared to the WT‐HGHP group. P‐RIP3 was significantly upregulated in the WT‐HGHP group, but remained low in ZBP1‐KO cells (Figure 4G‐H). These findings suggest that ZBP1 KO reduces necrosis and apoptosis in Tregs under HGHP. Western blot results confirmed that HGHP treatment upregulated p‐MLKL and cleaved caspase‐3 in WT Tregs. ZBP1‐KO‐HGHP Tregs showed significantly lower levels of p‐MLKL and cleaved caspase‐3 than the WT‐HGHP group, indicating that ZBP1 deficiency alleviates HGHP‐induced necrosis and apoptosis. Furthermore, western blot analysis confirmed that HGHP exposure enhanced the expression of the necroptosis marker p‐MLKL and the apoptosis marker cleaved caspase‐3 in WT Tregs. In contrast, ZBP1 deficiency significantly reduced HGHP‐induced upregulation of p‐MLKL and cleaved caspase‐3 (Figure 4I–K). In summary, ZBP1 deficiency protects Tregs from apoptosis and necroptosis, stabilizing their population under HGHP conditions.

**FIGURE 4:**
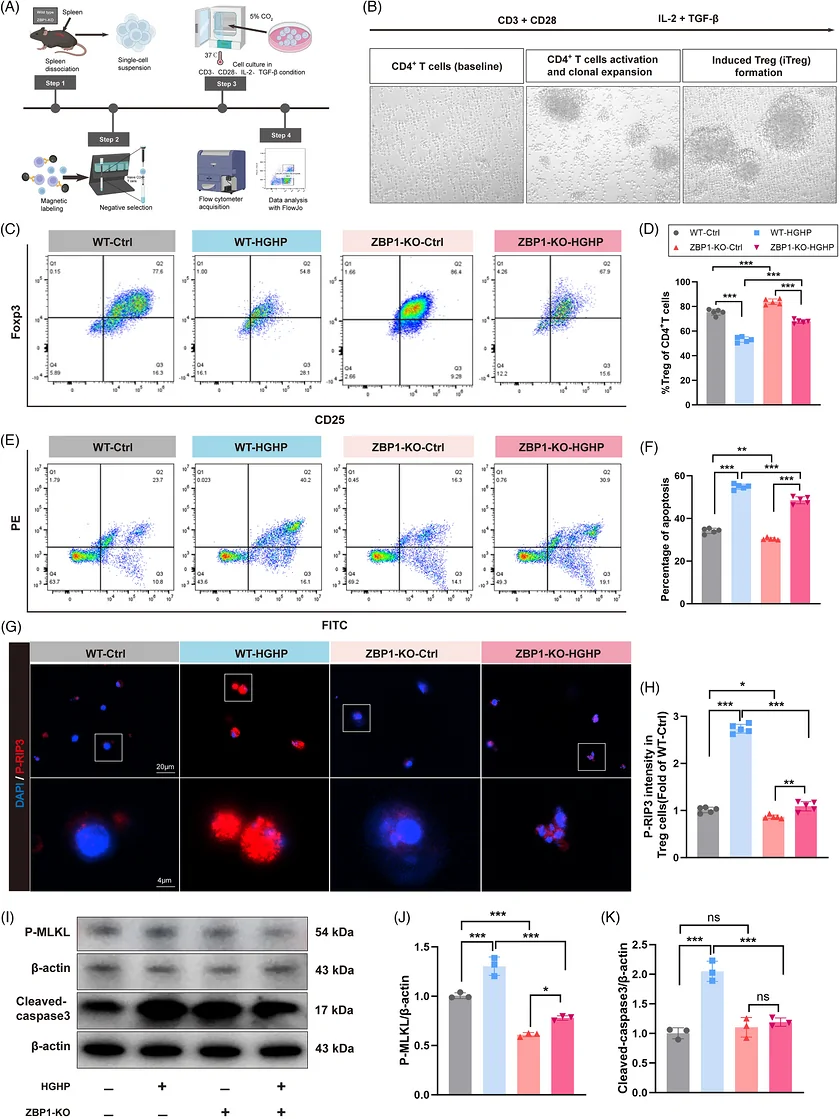
ZBP1 deficiency preserves Treg stability and limits HGHP‐induced apoptotic and necroptotic signaling. A, Schematic of the experimental workflow for splenic CD4^+^ T‐cell isolation, in vitro induction of Tregs, and downstream flow‐cytometric analyses. B, Representative bright‐field images showing CD4^+^ T cells at baseline, after activation and clonal expansion with CD3 and CD28, and after induction of Treg differentiation in the presence of IL‐2 and TGF‐β. C, Representative flow cytometry plots and quantification (D) of CD4^+^ CD25^+^ Foxp3^+^ Tregs derived from WT and ZBP1‐KO mice under control or HGHP conditions. E, Representative apoptosis plots and (F) quantification of apoptotic cells in the indicated groups (*n =* 5 per group). G, Representative immunofluorescence images and (H) quantification of p‐RIPK3 in Tregs from WT and ZBP1‐KO cultures under control or HGHP conditions (*n =* 5 per group). Scale bars, 20 µm (top) and 4 µm (bottom). I–K, Representative immunoblots and densitometric quantification of p‐MLKL and cleaved caspase‐3 in WT and ZBP1‐KO Tregs under the indicated conditions (*n =* 3 per group). Data are presented as mean ± standard deviation; NS, not significant; *P < 0.05, **P < 0.01, ***P < 0.001. HGHP, high glucose and high palmitate; IL, interleukin; KO, knockout; MLKL, mixed lineage kinase domain‐like; RIPK3, receptor interacting serine/threonine kinase 3; TGF‐β, transforming growth factor beta; Treg, regulatory T cell; WT, wild type; ZBP1, Z‐DNA‐binding protein 1.

### 3.5 ZBP1 deficiency attenuates T‐cell receptor signaling dysregulation and preserves Treg suppressive function under metabolic stress

We investigated the mechanism by which ZBP1 regulates Treg function. Immunofluorescence staining showed that ZBP1 co‐localized with LCK, a key component of T‐cell receptor (TCR) signaling (Figure 5A). Co‐immunoprecipitation experiments showed an interaction between ZBP1 and LCK (Figure 5B). Molecular docking analysis predicted defined interaction interfaces between ZBP1 and LCK (Figure 5C, D). Ramachandran plot analysis of ZBP1, LCK, and the ZBP1–LCK complex showed favorable backbone dihedral angle distributions, supporting stable protein conformations. In the predicted ZBP1–LCK complex, ZBP1 residues ARG115, ASP119, GLN107, and SER106 formed hydrogen bonds with LCK, which may contribute to stabilization of the complex. Together, these data suggest that ZBP1 interacts with LCK and may participate in the modulation of TCR signaling. We next examined TCR signaling activity under HGHP conditions. HGHP treatment markedly increased phosphorylation of LCK, zeta‐chain‐associated protein kinase 70 (ZAP70), and linker of activated T cells (LAT) in WT Tregs (Figure 5E–N). In contrast, ZBP1‐KO Tregs showed reduced levels of p‐LCK, p‐ZAP70, and p‐LAT under HGHP conditions, indicating that ZBP1 contributes to HGHP‐induced activation of the TCR signaling (Figure 5E–N). To determine the functional consequence of these signaling changes, we performed a CFSE‐based T‐cell suppression assay (Figure 5O). ZBP1‐KO‐HGHP Tregs exhibited stronger suppression of Tconv proliferation than WT‐HGHP Tregs, suggesting that ZBP1 deficiency preserves Treg‐mediated suppressive function under HGHP‐induced metabolic stress (Figure 5O, P). To further assess the functional state of Tregs, we measured cytokine concentrations in conditioned media collected from equal numbers of WT and ZBP1‐deficient Tregs cultured with or without HGHP. HGHP reduced IFN‐γ and IL‐10 concentrations and increased tumor necrosis factor alpha (TNF‐α) concentrations in WT Treg‐conditioned media. Compared to the WT‐HGHP group, the ZBP1‐KO‐HGHP group showed higher IFN‐γ and IL‐10 concentrations and lower TNF‐α concentrations (Figure S8A–C in supporting information). Collectively, these findings suggest that ZBP1 contributes to dysregulated proximal TCR signaling and impaired Treg function under glucolipotoxic stress, whereas ZBP1 deficiency preserves both Tconv‐suppressive activity and partially attenuates HGHP‐induced changes in cytokine levels in Treg‐conditioned media.

**FIGURE 5:**
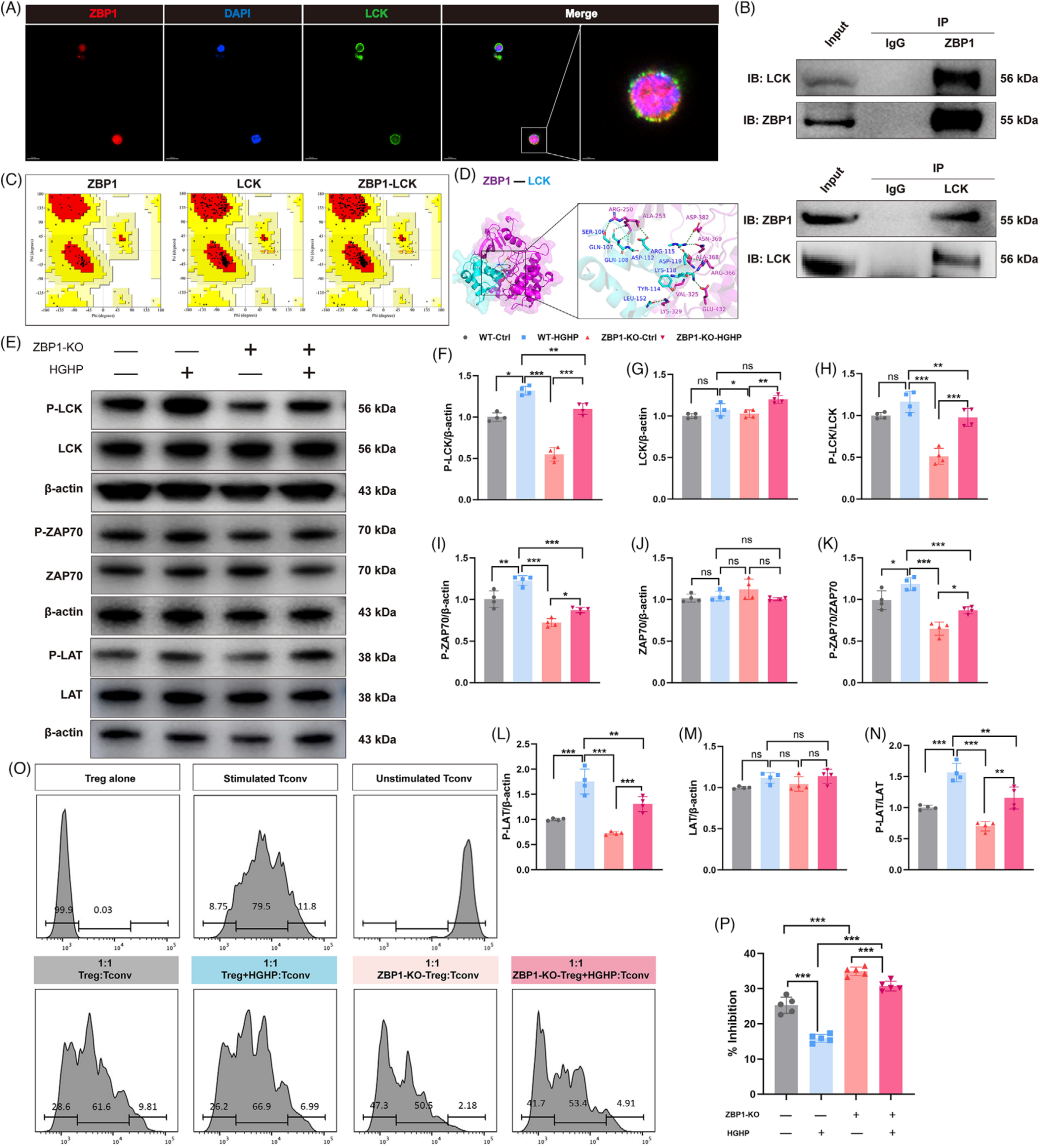
ZBP1 interacts with LCK and potentiates TCR signaling to impair Treg suppressive function under HGHP conditions. A, Representative immunofluorescence images showing co‐localization of ZBP1 and LCK in Tregs. Nuclei are counterstained with DAPI. B, Co‐immunoprecipitation analysis showing reciprocal interaction between ZBP1 and LCK in Tregs. C, D, Contact maps and structural modeling of the ZBP1‐LCK complex, showing the predicted interaction interface and representative binding residues. E, Representative immunoblots of p‐LCK, LCK, p‐ZAP70, ZAP70, p‐LAT, and LAT in WT and ZBP1‐KO Tregs under control or HGHP conditions. β‐actin serves as a loading control. F–N, Densitometric quantification of p‐LCK, LCK, p‐LCK/LCK, p‐ZAP70, ZAP70, p‐ZAP70/ZAP70, p‐LAT, total LAT, and p‐LAT/LAT (*n =* 4 per group). O, Representative CFSE dilution profiles of Tconv cells cultured alone or co‐cultured with WT or ZBP1‐KO Tregs under control or HGHP conditions. P, Quantification of Treg‐mediated inhibition of Tconv proliferation in the indicated groups (*n =* 5 per group). Data are presented as mean ± standard deviation; NS, not significant; *P < 0.05, **P < 0.01, ***P < 0.001. CFSE, carboxyfluorescein succinimidyl ester; DAPI, 4′,6‐diamidino‐2‐phenylindole; HGHP, high glucose and high palmitate; KO, knockout; LAT, linker of activated T cells; Tconv, conventional T cells; TCR, T‐cell receptor; Treg, regulatory T cell; WT, wild type; ZAP70, zeta‐chain‐associated protein kinase 70; ZBP1, Z‐DNA‐binding protein 1.

### 3.6 ZBP1 deficiency enhances Treg‐mediated suppression of microglial and astrocytic activation under metabolic stress

To determine whether ZBP1 regulates the immunomodulatory effects of Tregs on microglia, we established a transwell co‐culture system in which primary microglia or astrocytes were stimulated with Aβ_1‐42_ in the lower chamber and co‐cultured with or without Tregs in the upper chamber (Figure 6A). Immunoblot analysis showed that ZBP1 deficiency enhanced the capacity of Tregs to restrain Aβ_1‐42_‐induced microglial pro‐inflammatory activation under HGHP stress (Figure 6B, C). Consistently, immunofluorescence analysis showed that Aβ_1‐42_ markedly increased inducible nitric oxide synthase (iNOS) expression in primary microglia, whereas co‐culture with Tregs attenuated this response. HGHP exposure weakened the ability of WT Tregs to suppress iNOS induction. In contrast, ZBP1‐deficient Tregs exerted stronger inhibition of microglial iNOS expression, and this effect was partially maintained under HGHP conditions (Figure 6D‐F). We next examined whether ZBP1 in Tregs regulates microglial phagocytic activity using flow cytometry (Figure 6G). LPS/IFN‐γ stimulation markedly increased the uptake of fluorescent beads by microglia, whereas co‐culture with Tregs significantly suppressed this response. Notably, HGHP exposure attenuated the inhibitory effect of Tregs on microglial phagocytosis. In contrast, ZBP1‐deficient Tregs more effectively restrained microglial phagocytic activity, and this regulatory effect was partially maintained under HGHP conditions (Figure 6H,I). Additionally, Aβ_1‐42_ stimulation markedly increased C3 expression in primary astrocytes, indicative of A1‐like astrocyte activation, whereas co‐culture with Treg cells partially reduced C3 intensity. Notably, HGHP exposure attenuated the protective effect of Tregs and exacerbated astrocytic activation, while ZBP1‐deficient Tregs more effectively suppressed A1‐like astrocyte activation (Figure 6J–L). Collectively, these findings indicate that ZBP1 impairs Treg‐mediated control of microglial and astrocytic activation and phagocytic activity under metabolic stress.

**FIGURE 6:**
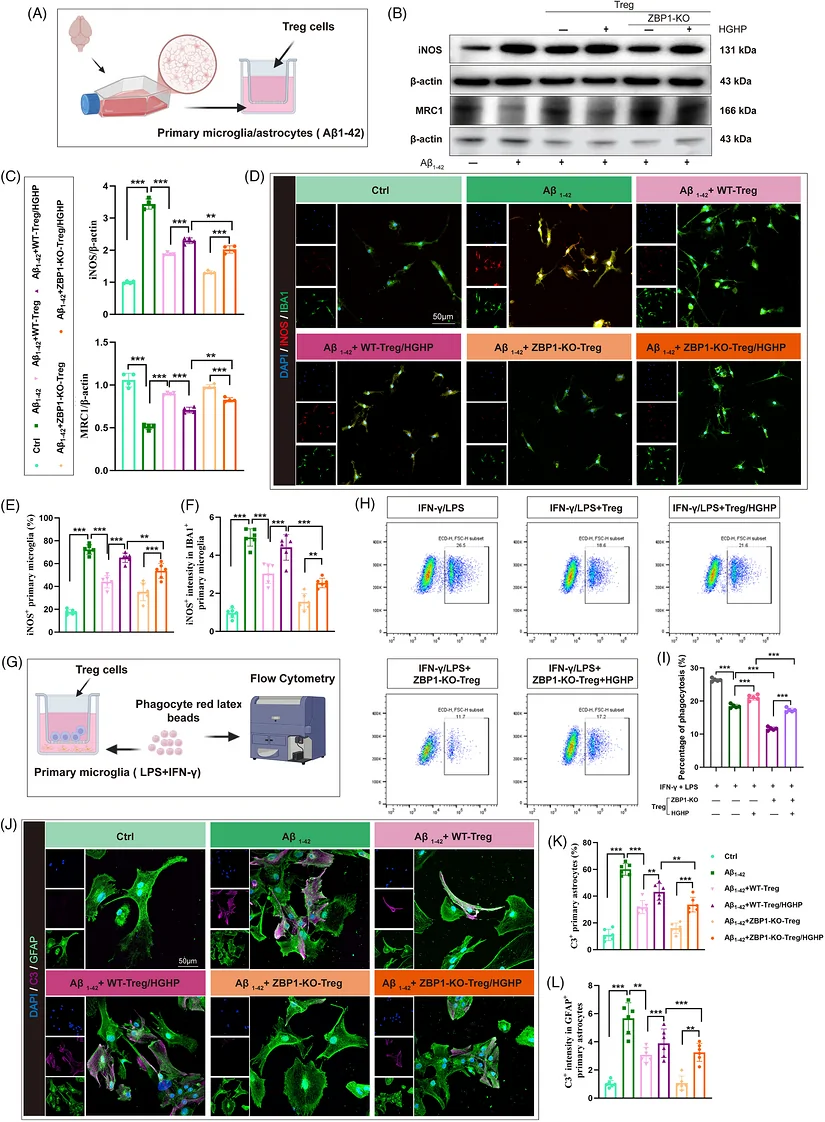
ZBP1 impairs Treg‐mediated suppression of microglial activation, phagocytic dysfunction, and astrocytic C3 activation under metabolic stress. A, Schematics of the transwell co‐culture systems used to assess the effects of Tregs on primary microglia and astrocytes. B, C, Representative immunoblots and densitometric quantification of iNOS and MRC1 in primary microglia in each group (*n =* 4 per group). D, Representative IF images of primary microglia stained for IBA1 (green) and iNOS (red) under the indicated conditions. Nuclei are counterstained with DAPI. Scale bar, 50 µm. E, F, Quantification of the percentage of iNOS^+^ primary microglia and iNOS fluorescence intensity within IBA1^+^ microglia (*n =* 6 per group). G, Schematic of the latex bead phagocytosis assay in primary microglia stimulated with LPS plus IFN‐γ and co‐cultured with Tregs. H, Representative flow‐cytometric plots of fluorescent latex bead uptake by primary microglia after LPS plus IFN‐γ stimulation in the presence or absence of WT or ZBP1‐KO Treg cells, with or without HGHP exposure. I, Quantification of the percentage of phagocytic microglia in the indicated groups (*n =* 5 per group). J, Representative IF images of primary astrocytes stained for GFAP (green) and C3 (purple) under the indicated conditions. Nuclei were counterstained with DAPI. Scale bar, 50 µm. K, L, Quantification of the percentage of C3+ astrocytes and C3 fluorescence intensity within GFAP+ astrocytes (*n =* 6 per group). Data are presented as mean ± standard deviation; NS, not significant; *P < 0.05, **P < 0.01, ***P < 0.001. Aβ, amyloid beta; DAPI, 4′,6‐diamidino‐2‐phenylindole; GFAP, glial fibrillary acidic protein; HGHP, high glucose and high palmitate; IBA1, ionized calcium‐binding adapter molecule 1; IF, immunofluorescence; IFN‐γ; interferon gamma; iNOS, inducible nitric oxide synthase; KO, knockout; LPS, lipopolysaccharide; MRC1, mannose receptor 1; Treg, regulatory T cell; WT, wild type; ZBP1, Z‐DNA‐binding protein 1.

### 3.7 ZBP1 deficiency preserves Treg‐mediated protection against neuronal injury under metabolic stress

Given the enhanced control of microglial and astrocytic activation by ZBP1‐deficient Tregs, we next assessed whether this effect extended to neuronal injury. In primary neurons, Aβ_1‐42_ disrupted neuronal morphology and synaptic integrity, as shown by reduced microtubule associated protein 2 (MAP2) and synaptophysin (SYN) signals (Figure 7A). WT Tregs partially mitigated these changes, whereas HGHP impaired this protection. ZBP1‐deficient Tregs better preserved MAP2 and SYN expression under HGHP stress (Figure 7B, C). Consistently, Aβ_1‐42_ induced mitochondrial depolarization in primary neurons, reflected by a reduced red/green fluorescence ratio. WT Tregs partially restored mitochondrial membrane potential, whereas HGHP weakened this effect. ZBP1‐deficient Tregs maintained a higher JC‐1 red/green ratio under HGHP conditions, indicating improved preservation of mitochondrial function (Figure 7C, D). Furthermore, Aβ_1‐42_ increased BCL2 associated X (BAX) and reduced BCL2 apoptosis regulator (BCL‐2) expression, indicating activation of pro‐apoptotic signaling. This response was attenuated by WT Tregs but re‐emerged under HGHP conditions. ZBP1‐deficient Tregs more strongly suppressed BAX induction and restored BCL‐2 levels (Figure 7E–G). Together, these findings indicate that ZBP1 deficiency sustains the neuroprotective capacity of Tregs against Aβ‐induced neuronal injury and mitochondrial dysfunction under metabolic stress.

**FIGURE 7:**
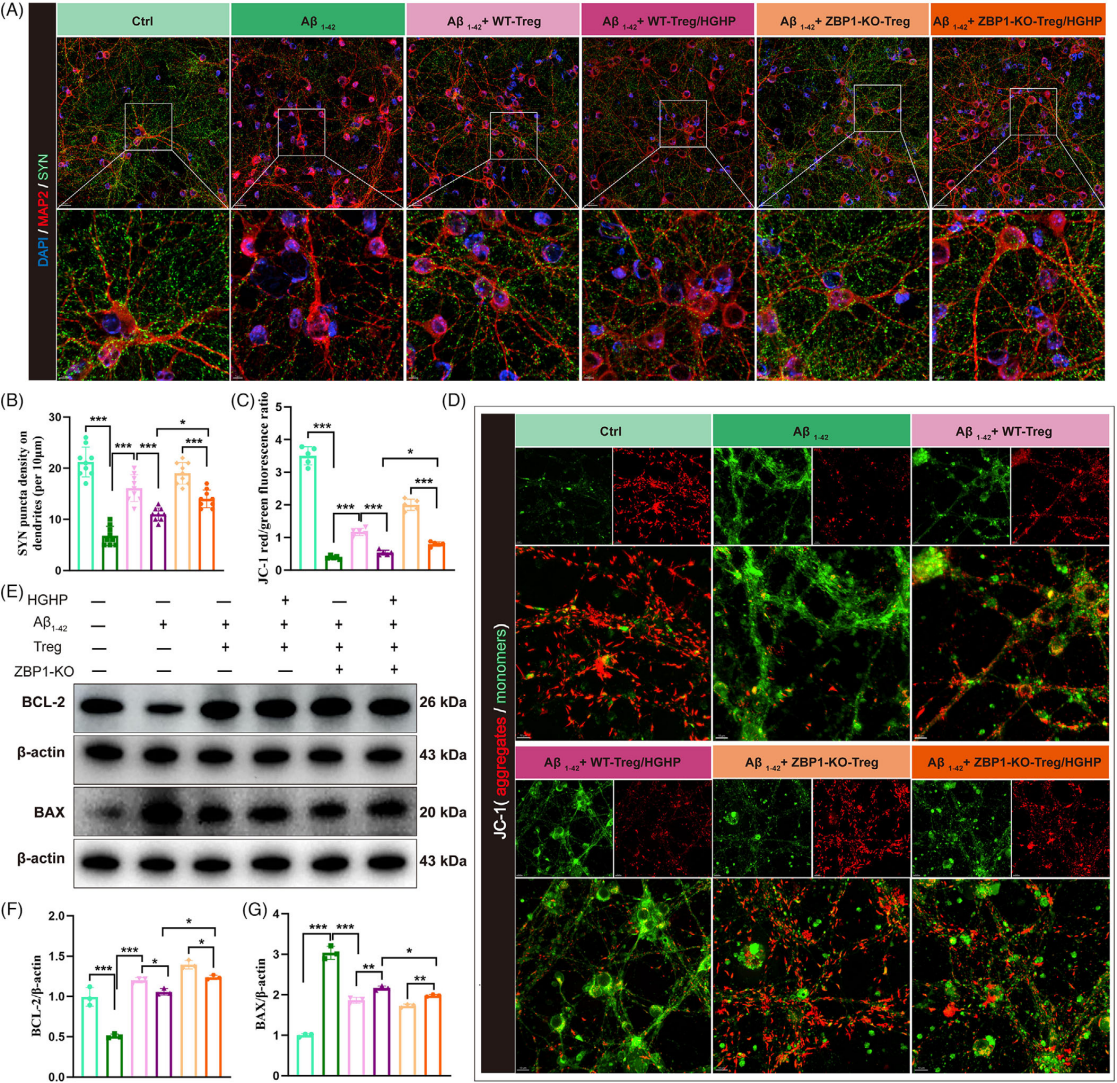
ZBP1 deficiency preserves Treg‐mediated protection against neuronal injury and mitochondrial dysfunction under HGHP conditions. A, Representative immunofluorescence images of primary neurons stained for MAP2 and SYN under the indicated conditions. Boxed regions are shown at higher magnification below. Scale bars: 20 µm (top) and 5 µm (bottom). Nuclei were counterstained with DAPI. B, Quantification of SYN puncta density along MAP2^+^ neurites (*n =* 9 per group). C, D, Quantification of the JC‐1 red/green fluorescence ratio in the indicated groups and representative JC‐1 fluorescence images showing mitochondrial membrane potential in primary neurons. Red fluorescence indicates JC‐1 aggregates, whereas green fluorescence indicates JC‐1 monomers (*n =* 5 per group). Scale bar, 20 µm. E–G, Representative immunoblots and quantification of BCL‐2 and BAX in primary neurons under the indicated conditions. β‐actin was used as the loading control (*n =* 3 per group). Data are presented as mean ± standard deviation; *P < 0.05, **P < 0.01, ***P < 0.001. Aβ, amyloid beta; BAX, BCL2 associated X; BCL‐2, BCL2 apoptosis regulator; DAPI, 4′,6‐diamidino‐2‐phenylindole; HGHP, high glucose and high palmitate; MAP2, microtubule associated protein 2; SYN, synaptophysin; Treg, regulatory T cell; WT, wild type; ZBP1, Z‐DNA‐binding protein 1.

### 3.8 ZBP1 deficiency potentiates the therapeutic efficacy of adoptively transferred Tregs in HFD‐fed AD mice

To determine whether ZBP1 deficiency enhances the therapeutic efficacy of Treg‐based intervention in vivo, we first optimized the adoptive transfer strategy using Tregs isolated from Foxp3^GFP^ reporter mice. PCR‐based genotyping confirmed Foxp3^GFP^ mice before Treg isolation, and green fluorescent protein–labeled Tregs were expanded in vitro and exposed to HGHP before transfer (Figure S9A, B in supporting information). Longitudinal flow cytometric analysis showed that adoptively transferred Foxp3^GFP^ Tregs remained detectable in AD‐HFD mice for several weeks but gradually declined over time (Figure S9C), providing the basis for the repeated transfer schedule used in Figure 8A. Accordingly, HGHP‐exposed WT or ZBP1‐deficient Tregs were adoptively transferred into HFD‐fed AD mice, followed by behavioral, imaging, histological, and electrophysiological analyses (Figure 8A). In the MWM, WT Treg transfer improved spatial learning and memory in AD‐HFD mice, as shown by reduced escape latency and first‐entry latency, increased platform crossings, and longer time spent in the target quadrant. These effects were further enhanced by ZBP1‐deficient Tregs (Figure 8B–F). Total swimming path length was comparable among groups (Figure 8G).

**FIGURE 8:**
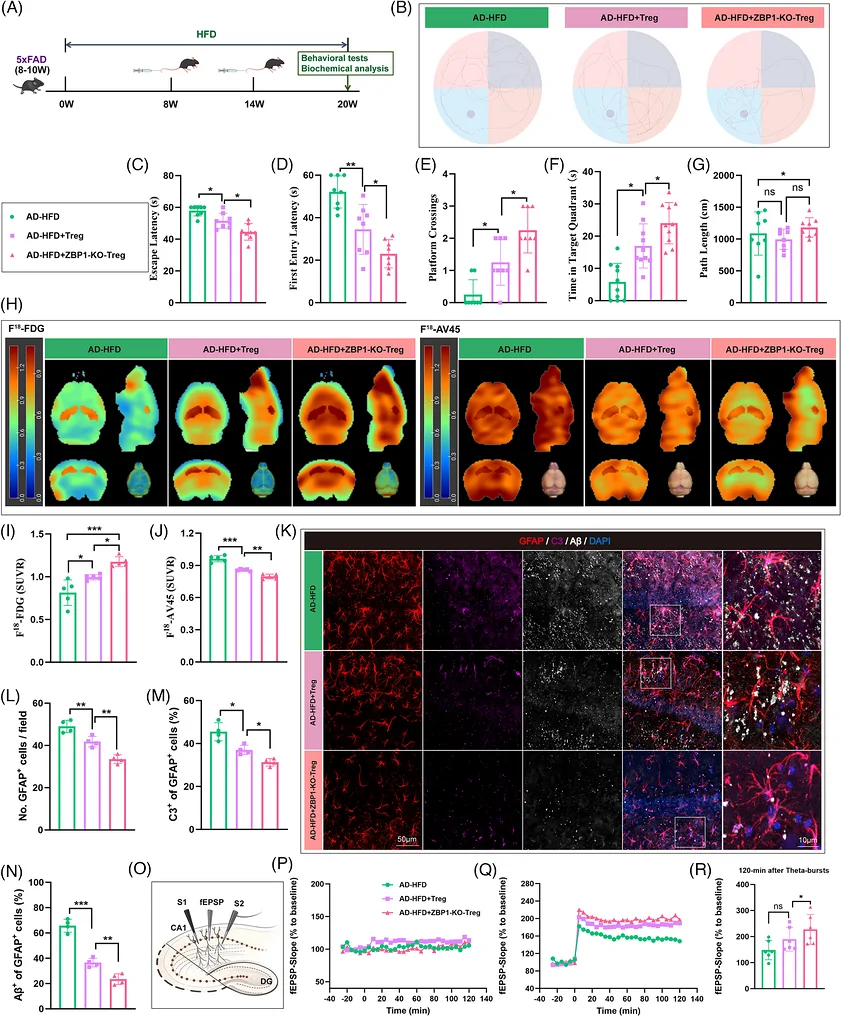
ZBP1‐deficient Tregs confer enhanced neuroprotection in HFD‐fed AD mice. A, Schematic of the in vivo experimental design. HFD‐fed AD mice received adoptive transfer of WT or ZBP1‐KO Tregs, followed by behavioral, PET imaging, histological, and electrophysiological analyses. B, Representative swimming trajectories from the MWM test in each group. Quantification of MWM performance, including (C) escape latency, (D) first‐entry latency, (E) platform crossings, (F) time spent in the target quadrant, and (G) path length (*n =* 8 per group). H, Representative F^18^‐FDG and F^18^‐AV45 PET images from the indicated groups. Quantification of hippocampal (I) F^18^‐FDG SUVR and (J) F^18^‐AV45 SUVR (*n =* 5 per group). K, Representative immunofluorescence images of hippocampal sections stained for GFAP, C3, Aβ, and DAPI. Boxed regions are shown at higher magnification. Scale bars, 50 and 10 µm. Quantification of GFAP^+^ cells per field (L), C3^+^GFAP^+^ astrocytes (M), and Aβ^+^ area (N) in the indicated groups (*n =* 4 per group). O, Schematic of the hippocampal recording configuration for fEPSP recordings at Schaffer collateral‐CA1 synapses. Time course of baseline fEPSP responses (P) and theta‐burst stimulation‐induced LTP (Q) in hippocampal slices in each group. R, Quantification of fEPSP slope at 120 minutes after theta‐burst stimulation (*n =* 6 per group). Data are presented as mean ± standard deviation; NS, not significant; *P < 0.05, **P < 0.01, ***P < 0.001. Aβ, amyloid beta; AD, Alzheimer's disease; AV45, florbetapir; DAPI, 4′,6‐diamidino‐2‐phenylindole; FDG, fluorodeoxyglucose; fEPSP, field excitatory postsynaptic potential; GFAP, glial fibrillary acidic protein; HFD, high‐fat diet; KO, knockout; LTP, long‐term potentiation; MWM, Morris water maze; PET, positron emission tomography; SUVR, standardized uptake value ratio; Treg, regulatory T cell; WT, wild type; ZBP1, Z‐DNA‐binding protein 1.

To evaluate hippocampal metabolic function and amyloid burden in each group, we performed ^18^F‐FDG and ^18^F‐AV45 PET imaging. Compared to AD‐HFD mice, mice receiving WT Tregs showed partial improvement in glucose use and a modest reduction in amyloid retention. Notably, transfer of ZBP1‐deficient Tregs resulted in a greater increase in FDG uptake and a more pronounced reduction in AV45 retention, indicating improved cerebral metabolism and reduced amyloid pathology (Figure 8H–J). Consistent with these findings, immunofluorescence analysis showed that AD‐HFD mice exhibited significant Aβ deposition, astrocyte responsiveness, and A1‐like activation, manifested as increased Aβ and GFAP expression, a higher proportion of C3^+^GFAP^+^ astrocytes. Transfusion of WT Tregs partially mitigated these changes, while ZBP1‐deficient Tregs more effectively suppressed astrocyte activation and A1 polarization (Figure 8K–M). Furthermore, mice receiving ZBP1‐deficient Tregs showed a further reduction in Aβ load compared to mice receiving WT Tregs (Figure 8N). To assess whether Treg transfer altered systemic and hippocampal inflammation, we measured serum cytokines and hippocampal inflammatory gene expression. WT Treg transfer increased serum IL‐10 and reduced serum TNF‐α while also decreasing hippocampal IL‐1β and TNF‐α mRNA expression, which were more pronounced with ZBP1‐deficient Tregs (Figure S10A–D in supporting information). These findings indicate that ZBP1 deficiency enhances the ability of transferred Tregs to attenuate both peripheral and hippocampal inflammatory responses. Across the three groups, hippocampal ^18^F‐FDG SUVR was inversely correlated with the proportion of C3^+^GFAP^+^ astrocytes (Figure S11A in supporting information). Field potential recordings showed that long‐term potentiation (LTP) was markedly impaired in AD‐HFD mice, while WT Treg transfer partially restored synaptic plasticity. Importantly, ZBP1‐deficient Tregs produced a greater rescue of LTP responses, indicating superior preservation of hippocampal synaptic function (Figure 8O–R). In addition, hippocampal LTP magnitude was inversely correlated with escape latency and positively correlated with platform crossings (Figure S11B, C). Together, these findings indicate that ZBP1 deficiency potentiates the therapeutic efficacy of adoptively transferred Tregs, improving cognition, cerebral metabolism, amyloid pathology, astrocyte reactivity, and hippocampal synaptic plasticity in HFD‐fed AD mice.

## 4 DISCUSSION

AD is increasingly recognized as a systemic disorder in which peripheral metabolic and immune perturbations critically shape disease progression.^32^, ^33^ In this study, metabolic stress increased ZBP1 expression in Tregs and was associated with activation of cell death–related signaling and impaired immunosuppressive function. ZBP1 deficiency preserved Treg survival and suppressive capacity and enhanced the protective effects of adoptively transferred Tregs in HFD‐fed 5xFAD mice. These findings identify ZBP1 as one component of the broader inflammatory and cellular stress network that contributes to metabolic stress–induced Treg dysfunction and AD‐related neuroimmune dysregulation.

Accumulating evidence supports a significant association between metabolic dysfunction and both the risk and progression of dementia. Large‐scale epidemiological studies have identified T2DM,^34^ obesity,^35^ and metabolic syndrome^36^ as major risk factors for dementia onset and progression. Importantly, the magnitude of this association appears to depend on the timing and duration of metabolic exposure, with midlife obesity, earlier onset diabetes, and longer diabetes duration being associated with a greater subsequent dementia risk.^37^, ^38^ These observations suggest that metabolic dysfunction may influence brain vulnerability during a prolonged preclinical period rather than acting only near the onset of cognitive symptoms. Persistent metabolic disturbances can disrupt systemic homeostasis and increase susceptibility to chronic disease.^39^ Previous studies have shown that obesity reduces metabolically protective Treg populations in adipose tissue and impairs Treg abundance and function in both experimental models and humans.^40^ Moreover, obesity‐associated immune and cellular phenotypes may persist even after weight reduction,^41^ supporting the broader concept that prolonged metabolic exposure can leave durable immunometabolic imprints.

HFD feeding accelerates Aβ deposition, amplifies neuroinflammatory responses, impairs synaptic plasticity, and precipitates cognitive decline,^8^, ^42^, ^43^ with immune dysregulation increasingly recognized as an important mediator of these effects.^44^ Obesity also remodels peripheral T‐cell populations and promotes dysfunctional or exhaustion‐like immune phenotypes,^45^, ^46^ indicating that metabolic stress can durably alter immune homeostasis. CD8^+^ T cells may accumulate in CNS‐associated compartments and contribute to persistent neuroinflammation, whereas disruption of the Th17/Treg balance may further favor pro‐inflammatory immune responses.^14^, ^16^, ^19^ Monocytes and macrophages may influence cytokine production and Aβ handling, while B cells may contribute through antigen presentation, cytokine secretion, and antibody‐mediated mechanisms.^13^ These immune populations may interact with Tregs and contribute to the neuroimmune effects of metabolic stress. Taken together, these observations suggest that prolonged metabolic stress may progressively disrupt innate and adaptive immune homeostasis and increase the vulnerability of the aging brain to AD‐related neuroinflammation and neurodegeneration. In this study, although the HFD‐fed 5xFAD model compresses the prolonged course of metabolic exposure in humans, it provides a tractable system for examining how sustained metabolic stress modifies an amyloid‐prone neuroinflammatory background.

Tregs are essential regulators of immune homeostasis, and their protective roles in neurodegenerative and neuroinflammatory disorders have received increasing attention.^47^, ^48^ Tregs may influence CNS inflammation through peripheral immune regulation, meningeal and CNS‐border compartments, and, in some disease contexts, direct entry into the CNS. Through these mechanisms, they can restrain excessive microglial and astrocytic activation, support tissue repair, and limit neurotoxic inflammatory responses.^48^, ^49^ In AD models, Treg expansion has been shown to attenuate microglia‐driven neuroinflammation, ameliorate Aβ burden, and rescue cognitive deficits.^15^ Previous studies have shown that metabolic stress reduces Treg abundance and dampens their suppressive function;^50^ however, the molecular underpinnings of these functional alterations remain largely elusive. We found that ZBP1 as a candidate regulator of Tregs under metabolic stress. ZBP1 has been established as a pivotal sensor that triggers RIPK3‐mediated PANoptosis,^51^ a coordinated inflammatory cell death integrating apoptosis, necroptosis, and pyroptosis.^52^, ^53^ A recent study showed that ZBP1‐driven necroptosis has been implicated in high glucose‐induced renal tubular injury.^54^ Furthermore, metabolic stress can engage a broad network of inflammatory and cellular stress pathways, including inflammasome activation, endoplasmic reticulum (ER) stress, oxidative and mitochondrial injury, impaired autophagy, and regulated cell death.^55^, ^56^ Other studies indicate that interferon‐activated JAK/STAT signaling can induce ZBP1 expression, while interferon regulatory factor 1 contributes to ZBP1 induction and inflammatory cell‐death signaling.^57^, ^58^ In parallel, oxidative stress‐induced mitochondrial DNA release and mitochondrial genome instability can generate endogenous nucleic acid signals sensed by ZBP1.^59^ ZBP1 may therefore amplify inflammatory cell death signals upon sensing these stress responses.

Beyond regulated cell‐death signaling, our findings suggest that ZBP1 is associated with modulation of the TCR signaling pathway, thereby altering Treg function. Although ZBP1 has not been previously reported as a regulator of TCR signaling, growing evidence indicates that innate immune pathways can influence T‐cell activation and function through crosstalk with TCR signaling,^60^, ^61^ providing a potential link between stress sensing and adaptive immune regulation. For instance, cellular stress sensors and metabolic checkpoints are recognized as interfacing with proximal TCR signaling networks, coupling metabolic reprogramming to T‐cell lineage commitment and functional specialization.^62^ We found that ZBP1 interacts with LCK and enhances activation of the LCK‐ZAP70‐LAT axis under HGHP conditions. ZBP1 may link glucolipotoxic stress to altered proximal TCR signaling and impaired Treg suppressive function.^63^ The stronger ability of ZBP1‐deficient Tregs to restrain microglial activation, C3‐associated astrocyte reactivity, and neuronal synaptic and mitochondrial injury further supports a model in which metabolic stress compromises peripheral immune regulation and amplifies neuroinflammatory damage in AD. Although these loss‐of‐function findings support a contributory role for ZBP1, complementary studies involving ZBP1 overexpression in WT Tregs and controlled re‐expression in ZBP1‐deficient Tregs will be needed to establish sufficiency and rescue, respectively.

Our findings also further support the potential of Treg‐based immunotherapy in metabolically impaired AD. Previous studies have shown that expansion or adoptive transfer of Tregs can reduce neuroinflammation and improve outcomes in models of neurodegeneration and neuroinflammation.^15^, ^64^ Intravenously transferred Tregs may act through peripheral immune regulation, CNS border compartments, or subsequent localization within the CNS, and these mechanisms may vary with disease context and time after transfer.^22^, ^65^ In the present study, adoptive transfer of WT Tregs partially attenuated AD‐related pathology, whereas ZBP1‐deficient Tregs conferred greater protection. These findings indicate that intrinsic metabolic stress state of transferred Tregs influences their immunoregulatory activity in vivo and, consequently, the efficacy of adoptive Treg therapy. Synaptic plasticity is a central substrate of learning and memory, and its preservation is increasingly recognized as an important determinant of cognitive resilience during aging and neurodegeneration.^66^ In our study, ZBP1‐deficient Treg transfer produced a greater restoration of synaptic potentiation than WT Treg transfer. In this context, our results support modulating Treg‐intrinsic ZBP1 rather than inhibiting ZBP1 systemically. ZBP1 functions as an innate nucleic acid sensor during viral infection and coordinates infection‐induced regulated cell‐death responses.^23^, ^67^, ^68^ Chronic systemic inhibition of ZBP1 could therefore compromise antiviral host defense. Treg‐restricted, transient, or ex vivo modulation may provide a more selective strategy for preserving Treg function while limiting effects on other immune‐cell populations. Collectively, these findings suggest that Treg‐intrinsic ZBP1 modulation warrants further investigation as a potential approach to preserve Treg function under metabolic stress and enhance the effects of adoptive Treg transfer in AD models.

This study has several limitations. First, the clinical component was cross‐sectional, involved a relatively small sample, and relied on cognitive screening instruments rather than comprehensive neuropsychological assessment. AD‐specific biomarkers, neuroimaging, prospectively collected peripheral blood mononuclear cells, and cerebrospinal fluid were unavailable. Accordingly, the human data should be interpreted as exploratory clinical evidence linking metabolic dysfunction with cognitive impairment. Although the analyses adjusted for major demographic and vascular–metabolic covariates, residual confounding remains possible, particularly because T2DM duration and long‐term glycemic control were not systematically recorded. Future prospective studies should incorporate AD‐related biomarkers, structural or functional neuroimaging, and dedicated biospecimen collection to evaluate circulating Treg phenotype, ZBP1 expression, and Treg function in relation to metabolic status and cognitive outcomes. Second, the current study relies primarily on genetic loss‐of‐function evidence. ZBP1 re‐expression and controlled gain‐of‐function studies are needed to establish rescue and sufficiency. Future studies should also determine whether selective pharmacological inhibition or targeted degradation of ZBP1 can reproduce the protective effects of genetic deletion in Tregs. Third, only male mice were included in the present study. Because sex can influence AD‐related pathology, metabolic responses, and immune regulation, the findings may not fully generalize to females. Finally, the HFD‐fed 5xFAD model mimics metabolic stress–exacerbated amyloid‐associated neuroimmune pathology but does not fully reflect the prolonged metabolic exposure, metabolic and pathological heterogeneity, and slow progression of sporadic late‐onset AD in humans. Future studies using slower progressing AD models, aging models, or diabetes models with longer metabolic exposure will be needed to determine whether the ZBP1–Treg axis contributes to the interaction between metabolic disease and sporadic AD pathogenesis.

In summary, our findings suggest that metabolic stress contributes to neuroimmune dysregulation partially through ZBP1‐associated impairment of Treg survival and suppressive function. ZBP1‐deficient Tregs showed greater stability and immunoregulatory activity under metabolic stress and conferred stronger protection in HFD‐fed AD mice. These preclinical findings support further investigation of Treg‐intrinsic ZBP1 modulation, but validation in human tissues and longitudinal cohorts will be required to establish its clinical relevance and translational potential.

## CONFLICT OF INTEREST STATEMENT

The authors declare no conflicts of interest. Author disclosures are available in the Supporting Information.

## CONSENT STATEMENT

The study involving human participants has been approved by the ethics committee of Huashan Hospital affiliated to Fudan University (KY2018‐250:01) and complies with the Declaration of Helsinki and its subsequent revisions. All animal experiments were performed in accordance with the National Institutes of Health Guidelines for the Care and Use of Laboratory Animals and approved by the Department of Laboratory Animal Science, Fudan University (Approval No. 202407015S).

## Supporting information




**Supporting Information**: alz71896‐sup‐0001‐SuppMat.docx

**Supporting Information**: alz71896‐sup‐0002‐ICMJE.pdf
