# Supplementary material for: ZBP1‐mediated Treg cell dysfunction exacerbates neuroimmune dysregulation in Alzheimer's disease under metabolic stress
Source: Alzheimers Dement. 2026 Sep 30;22(10):e71896. doi: 10.1002/alz.71896 (PMC13626149; doi:10.1002/alz.71896)
Supplement: Supplementary file 2 [file ALZ-22-e71896-s002.docx]

**ZBP1-mediated Treg cells dysfunction exacerbates neuroimmune dysregulation in Alzheimer’s disease under metabolic stress**

Shanshan Huang^1,2,3#^, Zengyu Zhang^4#^, Jiajie Xia^5#^, Mengqiu Deng^6^, Fangzheng Chen^2^, Meng Wang^1,2^, Yuchao Fei^5^, Zhiwen Jiang^5^, Chenyu Lu^2^, Jiayu Fu^1^, Jingchun Guo^2*^, Houguang Zhou^1*^

^1^ Department of Geriatrics, Huashan Hospital, National Clinical Research Center for Aging and Medicine, Fudan University, Shanghai, China;

^2^ MOE Frontiers Center for Brain Science, Institutes of Brain Science, Department of Translational Neuroscience, Jing'an District Centre Hospital of Shanghai, Fudan University, Shanghai, China;

^3^ Department of Endocrinology, Affiliated Hospital of Jiangnan University, Jiangnan University, Wuxi, China;

^4^ Department of Rehabilitation Medicine, Huashan Hospital, Fudan University, Shanghai, China;

^5^ Department of Neurosurgery, Huashan Hospital, Fudan University, Shanghai, China.

^6^ Department of Anesthesiology, Changzheng Hospital, Second Affiliated Hospital of Naval Medical University, Shanghai, China

**#The authors contributed equally to this study.**

***Correspondence**

Houguang Zhou, Department of Geriatrics, Huashan Hospital, National Clinical Research Center for Aging and Medicine, Fudan University, Shanghai, 200040, China Email: zhouhouguang@huashan.org.cn; zhg7376@163.com

Jingchun Guo, MOE Frontiers Center for Brain Science, Institutes of Brain Science, Department of Translational Neuroscience, Jing'an District Centre Hospital of Shanghai, Fudan University, Shanghai, 200032, China

Email: [jingchunguo@shmu.edu.cn](mailto:jingchunguo@shmu.edu.cn)

**Table S1. Antibodies for western blotting and IF analysis**

| Target | Supplier | Catalog# | Host (raised in) |
| --- | --- | --- | --- |
| BACE1 | Cell Signaling Technology | 5606 | Rabbit |
| NF-L | Abcam | ab223343 | Rabbit |
| PSD95 | Cell Signaling Technology | 3450 | Rabbit |
| GFAP | Cell Signaling Technology | 3670S | Mouse |
| ZBP1 | Santa Cruz | sc-271483 | Mouse |
| Foxp3 | Cell Signaling Technology | 12653 | Rabbit |
| GSDMD | Cell Signaling Technology | 39754 | Rabbit |
| P-MLKL | Cell Signaling Technology | 37333 | Rabbit |
| C3 | Invitrogen | PA1-29715 | Goat |
| P-RIP3 | Cell Signaling Technology | 93654 | Rabbit |
| Cleaved-caspase3 | Cell Signaling Technology | 9661 | Rabbit |
| LCK | Cell Signaling Technology | 2752 | Rabbit |
| P-LCK | Cell Signaling Technology | 2751 | Rabbit |
| ZAP70 | Cell Signaling Technology | 3165 | Rabbit |
| P-ZAP70 | Cell Signaling Technology | 2717 | Rabbit |
| LAT | Cell Signaling Technology | 45533 | Rabbit |
| P-LAT | Cell Signaling Technology | 3584 | Rabbit |
| iNOS | Abcam | ab178945 | Rabbit |
| IBA1 | Abcam | ab283319 | Mouse |
| MRC1 | Cell Signaling Technology | 24595 | Rabbit |
| SYN | Abcam | Ab32127 | Rabbit |
| Beta(β)-Amyloid | Sigma-Aldrich | AB9234 | Rabbit |
| MAP2 | Cell Signaling Technology | 78667 | Mouse |
| BCL-2 | Cell Signaling Technology | 3948 | Rabbit |
| BAX | Cell Signaling Technology | 2774 | Rabbit |
| β-actin | Cell Signaling Technology | 3700S | Mouse |
| Anti-rabbit IgG | Cell Signaling Technology | 7074P2 | Goat |
| Anti-mouse IgG | Cell Signaling Technology | 7076P2 | Horse |
| Donkey anti-Rabbit IgG, Alexa Fluor™594 | Invitrogen | A-21207 | Donkey |
| Donkey anti-Rabbit IgG, Alexa Fluor™488 | Invitrogen | A-21206 | Donkey |
| Donkey anti-Mouse IgG, Alexa Fluor™488 | Invitrogen | A-21202 | Donkey |
| Donkey anti-Goat IgG, Alexa Fluor™647 | Invitrogen | A-21447 | Donkey |

**Table S2. Fluorophores for flow cytometry**

| Target | Supplier | Catalog# |
| --- | --- | --- |
| Brilliant Violet 605™ anti-mouse CD3 Antibody | BioLegend | 100237 |
| APC/Fire™ 750 anti-mouse CD4 Antibody | BioLegend | 100568 |
| Alexa Fluor® 700 anti-mouse CD8a Antibody | BioLegend | 100730 |
| BV421 Rat Anti-Mouse CD25 | BD Biosciences | 564571 |
| Zombie Aqua™ Fixable Viability Kit | BioLegend | 423102 |
| FOXP3 Monoclonal Antibody (FJK-16s), FITC | eBioscience | 11-5773-82 |
| TruStain FcX™ (anti-mouse CD16/32) Antibody | BioLegend | 101320 |

**Figure S1 Representative flow-cytometric gating strategy for the identification of Treg cells in peripheral blood and brain.**

Cells were initially selected according to forward- and side-scatter characteristics (A), followed by exclusion of doublets using FSC-A versus FSC-H gating (B). Viable cells were identified by exclusion of viability dye-positive events (C). CD3⁺ T cells were then selected (D), followed by identification of CD4⁺CD8⁻ T cells (E). Treg cells were defined as CD25⁺Foxp3⁺ cells within the viable CD3⁺CD4⁺CD8⁻ T-cell population (F).

**Table S3. Demographic and clinical characteristics of participants with and without T2DM.**

|  | **Total (n=290)** | **T2DM (n=102)** | **Non-T2DM (n=188)** | ***P*-value** |
| --- | --- | --- | --- | --- |
| **Age (year)** **(mean±SD)** | 78.69 ± 9.32 | 81.06 ± 9.33 | 77.39 ± 9.08 | 0.002 |
| **Male (n, %)** | 155 (53.45%) | 55 (53.92%) | 100 (53.19%) | 0.906 |
| **Education (n, %)** |  |  |  | 0.012 |
| **Illiteracy** | 18 (6.21%) | 11 (10.78%) | 7 (3.72%) |  |
| **Primary** | 55 (18.97%) | 26 (25.49%) | 29 (15.43%) |  |
| **Lower secondary** | 136 (46.90%) | 40 (39.22%) | 96 (51.06%) |  |
| **Upper secondary and higher** | 81 (27.93%) | 25 (24.51%) | 56 (29.79%) |  |
| **Height (cm) (mean±SD)** | 162.95 ± 7.51 | 162.97 ± 7.72 | 162.95 ± 7.42 | 0.940 |
| **Weight (kg) (mean±SD)** | 63.21 ± 9.47 | 63.83 ± 10.03 | 62.87 ± 9.17 | 0.442 |
| **Waist (cm) (mean±SD)** | 88.42 ± 11.28 | 87.57 ± 11.11 | 90.01 ± 11.48 | 0.059 |
| **BMI (kg/m²) (mean±SD)** | 23.75 ± 2.81 | 24.01 ± 3.30 | 23.61 ± 2.51 | 0.436 |
| **Hypertension (n, %)** | 195 (67.24%) | 82 (80.39%) | 113 (60.11%) | ＜0.001 |
| **Hyperlipidemia (n, %)** | 40 (13.79%) | 20 (19.61%) | 20 (10.64%) | 0.053 |
| **MMSE (median, IQR)** | 23 (19 - 24) | 21 (14 - 24) | 23 (20 - 24) | ＜0.001 |
| **SCD-Q9** | 5 (2.5 – 6.5) | 5.5 (3-7.5) | 4.5 (2-6.5) | ＜0.001 |

BMI, Body mass index; IQR, Interquartile range; MMSE, Mini-Mental State Examination; SCD-Q9, Subjective Cognitive Decline Questionnaire of 9-item

**Table S4. Association of T2DM with MMSE score.**

|  | ***β*** | **95% CI** | ***P*-value** |
| --- | --- | --- | --- |
| **Model 1** | -3.86 | -5.50, -2.23 | ＜0.001 |
| **Model 2** | -2.96 | -4.40, -1.52 | ＜0.001 |
| **Model 3** | -2.15 | -3.19, -1.10 | ＜0.001 |
| **Model 4** | -2.31 | -3.38, -1.24 | ＜0.001 |

MMSE, Mini-Mental State Examination; BMI, body mass index; CI, confidence interval.

Effect estimates are presented as β coefficients with 95% confidence intervals from linear regression models. Model 1: unadjusted; Model 2: adjusted for age and sex; Model 3: adjusted for age, sex, and education; Model 4: adjusted for age, sex, and education, BMI, hypertension, and hyperlipidemia.

**Table S5. Association of T2DM with SCD-Q9 score.**

|  | ***β*** | **95% CI** | ***P*-value** |
| --- | --- | --- | --- |
| **Model 1** | 1.26 | 0.60, 1.91 | ＜0.001 |
| **Model 2** | 0.98 | 0.35, 1.61 | 0.002 |
| **Model 3** | 0.84 | 0.24, 1.43 | 0.006 |
| **Model 4** | 0.71 | 0.09, 1.33 | 0.024 |

SCD-Q9, Subjective Cognitive Decline Questionnaire of 9-item; BMI, Body mass index; CI, confidence interval.

Effect estimates are presented as β coefficients with 95% confidence intervals from linear regression models. Model 1: unadjusted; Model 2: adjusted for age and sex; Model 3: adjusted for age, sex, and education; Model 4: adjusted for age, sex, and education, BMI, hypertension, and hyperlipidemia.

**Figure S2 Correlation between metabolic impairment and spatial memory performance in 5xFAD mice.**

(A-C) Scatter plots showing the relationship between IPGTT AUC and MWM performance in HFD-fed 5xFAD mice. (A) Escape latency, (B) first-entry latency, and (C) number of platform crossings. (D-F) Scatter plots showing the relationship between ITT AUC and MWM performance in HFD-fed 5xFAD mice. (D) Escape latency, (E) first-entry latency, and (F) number of platform crossings. Pearson correlation coefficients (R) and p-values are indicated. Linear regression lines are shown in black, with 95% confidence intervals indicated by dotted lines.

**Figure S3 Correlation between metabolic impairment and spontaneous alternation and locomotor activity in 5xFAD mice**

(A, B) Scatter plots showing the relationship between IPGTT AUC and Y-maze performance. (A) Percentage of spontaneous alternation, (B) total movement speed. c, d, Scatter plots showing the relationship between ITT AUC and Y-maze performance. (C) Percentage of spontaneous alternation, (D) total movement speed. Pearson correlation coefficients R and *p*-values are indicated. Linear regression lines are shown in black, with 95% confidence intervals indicated by dotted lines.

**Figure S4 HFD exacerbates hippocampal microgliosis, astrogliosis, and dendritic spine loss in AD mice**

(A, C) Representative immunofluorescence images of hippocampal sections stained for IBA1 (microglia; red) (A) and GFAP (astrocytes; purple) (C) in WT and 5xFAD mice fed a CD or HFD. Nuclei were counterstained with DAPI. Boxed regions are shown at higher magnification. Scale bars, 50μm (B, D) Quantification of IBA1^+^ microglial area (B) and GFAP⁺ astrocytic area (D) in CA1, CA3, and DG subregions (n=4 per group). (E) Representative Golgi-stained images showing dendritic morphology in CA1 pyramidal neurons. Insets highlight dendritic spines in WT-CD, WT-HFD, AD-CD, and AD-HFD mice. Quantification of dendritic spine density (F) and the proportion of spine types (G) (mushroom, stubby, thin, filopodia) in CA1 neurons from the indicated groups (n=8 per group). Data are presented as mean ± SD; NS, not significant; **P* < 0.05, ***P* < 0.01, ****P* < 0.001.

**Figure S5** **ZBP1 expression across immune cell populations, including T-cell and Treg subsets**

(A, B) UMAP plots of single-cell transcriptomic data from the mouse hippocampus highlight T-cell populations, including Treg-like subsets. (C) A heatmap of normalized transcript expression (log2[nTPM+1], z-score) shows ZBP1 enrichment in T cells. (D) Immune-cell expression data from the Human Protein Atlas show ZBP1 expression across multiple immune populations, including T-cell and Treg subsets.

**Figure S6 ZBP1 mRNA expression in purified Treg cells.**

CD4^+^CD25^+^ Treg cells were purified from the spleens of WT-CD, WT-HFD, AD-CD, and AD-HFD mice. Z	BP1 mRNA expression was measured by qPCR, normalized to GAPDH, and expressed relative to the WT-CD group using the 2^−ΔΔCt^ method (n=5 per group). Data are presented as mean ± SD; NS, not significant; **P* < 0.05, ***P* < 0.01, ****P* < 0.001.

**Figure S7** **Gene identification of ZBP1-KO mice.**

**Figure S8. ZBP1 deficiency influences cytokine secretion by Tregs under HGHP conditions.**

Equal numbers of WT and ZBP1-KO Tregs were cultured with or without HGHP. Concentrations of (A) IFN-γ, (B) IL-10, and (C) TNF-α in Treg-conditioned media were measured by ELISA (n=8 per group). Data are presented as mean ± SD; NS, not significant; **P* < 0.05, ***P* < 0.01, ****P* < 0.001.

**Figure S9 Identification, ex vivo expansion, and peripheral persistence of Foxp3^GFP^ Tregs cells after adoptive transfer.**

(A) Representative PCR genotyping confirming the Foxp3^GFP^ allele. (B) Representative bright-field images showing the stepwise dissociation, clonal expansion, and differentiation of Foxp3^GFP^ Tregs before adoptive transfer. (C) Representative flow-cytometric plots showing donor-derived Foxp3^GFP^ Treg cells in peripheral blood at weeks 1-4 after tail-vein transfer into HFD-fed AD mice.

**Figure S10 Treg transfer attenuates peripheral and hippocampal inflammation in AD-HFD mice**

Serum IL-10 (A) and TNF-α (B) levels were measured by ELISA in each group. Hippocampal IL-1β (C) and TNF-α (D) mRNA levels were measured by qPCR (n=5 per group). Data are presented as mean ± SD; **P* < 0.05, ***P* < 0.01, ****P* < 0.001.

**Figure S11 Correlation analyses linking cerebral metabolism, neuroinflammation, synaptic plasticity, and cognitive performance after Treg-based intervention.**

(A) Correlation between hippocampal ^18^F-FDG SUVR and the proportion of C3⁺ cells among GFAP⁺ astrocytes across animals from the AD-HFD, AD-HFD+Treg, and AD-HFD+ZBP1-KO-Treg groups (n=12 in total). (B) Correlation between hippocampal LTP magnitude (normalized fEPSP slope after theta-burst stimulation) and escape latency in the Morris water maze. (C) Correlation between hippocampal LTP magnitude and platform crossings in the Morris water maze. Each point represents one animal. Pearson correlation analysis was performed.
